# Gasdermin D restricts *Burkholderia cenocepacia* infection in vitro and in vivo

**DOI:** 10.1038/s41598-020-79201-5

**Published:** 2021-01-13

**Authors:** Shady Estfanous, Kathrin Krause, Midhun N. K. Anne, Mostafa Eltobgy, Kyle Caution, Arwa Abu Khweek, Kaitlin Hamilton, Asmaa Badr, Kylene Daily, Cierra Carafice, Daniel Baetzhold, Xiaoli Zhang, Tianliang Li, Haitao Wen, Mikhail A. Gavrilin, Hesham Haffez, Sameh Soror, Amal O. Amer

**Affiliations:** 1 Department of Microbial Infection and Immunity, Infectious Diseases Institute, Ohio State University, Columbus, OH USA; 2 Biochemistry and Molecular Biology Department, Faculty of Pharmacy, Helwan University, Cairo, Egypt; 3 Max Planck Unit for the Science of Pathogens, Berlin, Germany; 4 Department of Biology and Biochemistry, Birzeit University, Birzeit, West Bank Palestine; 5 Center for Biostatistics, Ohio State University, Columbus, OH USA; 6 Department of Internal Medicine, Ohio State University, Columbus, OH USA; 7 Center for Scientific Excellence “Helwan Structural Biology Research” (HSBR), Helwan University, Cairo, Egypt

**Keywords:** Cell biology, Immunology, Microbiology, Molecular biology, Diseases

## Abstract

*Burkholderia cenocepacia* (*B. cenocepacia*) is an opportunistic bacterium; causing severe life threatening systemic infections in immunocompromised individuals including cystic fibrosis patients. The lack of gasdermin D (GSDMD) protects mice against endotoxin lipopolysaccharide (LPS) shock. On the other hand, GSDMD promotes mice survival in response to certain bacterial infections. However, the role of GSDMD during *B. cenocepacia* infection is not yet determined. Our in vitro study shows that GSDMD restricts *B. cenocepacia* replication within macrophages independent of its role in cell death through promoting mitochondrial reactive oxygen species (mROS) production. mROS is known to stimulate autophagy, hence, the inhibition of mROS or the absence of GSDMD during *B. cenocepacia* infections reduces autophagy which plays a critical role in the restriction of the pathogen. GSDMD promotes inflammation in response to *B. cenocepacia* through mediating the release of inflammasome dependent cytokine (IL-1β) and an independent one (CXCL1) (KC). Additionally, different *B. cenocepacia* secretory systems (T3SS, T4SS, and T6SS) contribute to inflammasome activation together with bacterial survival within macrophages. In vivo study confirmed the in vitro findings and showed that GSDMD restricts *B. cenocepacia* infection and dissemination and stimulates autophagy in response to *B. cenocepacia*. Nevertheless, GSDMD promotes lung inflammation and necrosis in response to *B. cenocepacia* without altering mice survival. This study describes the double-edged functions of GSDMD in response to *B. cenocepacia* infection and shows the importance of GSDMD-mediated mROS in restriction of *B. cenocepacia.*

## Introduction

*Burkholderia cenocepacia* (*B. cenocepacia*) is an opportunistic aerobic Gram-negative facultative intracellular bacterium widely spread in the environment and can cause severe pulmonary infections in immunocompromised individuals and patients with underlying diseases such as cystic fibrosis (CF) and chronic granulomatous disease^1^. Treating such infections is challenging due to the evolving antimicrobial resistance of *B. cenocepacia*^2^. The infection state can further deteriorate to chronic lung inflammation and a life-threatening systemic infection known as cepacia syndrome, that in turn significantly decreases the patients’ survival^1,3–5^. Discovering alternative treatment targets to alleviate inflammation and resolve *B. cenocepacia* infections is crucial to the treatment of these severe and resistant infections.

Inflammasome activation is a fundamental mechanism for macrophages to respond to pathogens. The inflammasome promotes the activation and release of pro-inflammatory cytokines interleukin-1beta (IL-1β) and IL-18. This is associated with the formation of pores within the host plasma membrane via the protein Gasdermin D (GSDMD) which often precedes cell death^6–9^. GSDMD is expressed as an inactive auto-inhibitory form called GSDMD-full length (GSDMD-FL) which releases its inhibitory C-terminal domain upon activation. The liberated active N-terminal domains (GSDMD-NT) oligomerize and incorporate into the plasma membrane to form transmembrane pores^10,11^. When the GSDMD pores are excessive and repair mechanisms fail, cell death occurs. GSDMD is activated by caspase1 (CASP1) and/or caspase-11 (CASP11) via the canonical or non-canonical inflammasome pathway, respectively^11^. Recently, our research team has demonstrated that CASP11 is stimulated upon *B. cenocepacia* infection which can activate CASP1 in murine macrophages^12,13^. CASP11 was found to play a critical role in the restriction of many Gram-negative bacteria, including *B. cenocepacia,* within macrophages^12,14^. Nonetheless, the role of GSDMD in controlling *B. cenocepacia* infection is not yet explored.

This study demonstrates that despite lacking a direct bactericidal effect, GSDMD inhibits *B. cenocepacia* infection, independent from cell death. Instead, GSDMD activation mediates mitochondrial reactive oxygen species (mROS) production, which in turn stimulates the cellular autophagic machinery to degrade the invading bacteria in macrophages. Additionally, different *B. cenocepacia* secretory systems (T3SS, T4SS, and T6SS) contribute to GSDMD activation and hence bacterial clearance within macrophages. Moreover, GSDMD restricts *B. cenocepacia* replication while promoting inflammation and neutrophil recruitment in the lungs of infected mice. These results clarify the double-edged functions of GSDMD in response to *B. cenocepacia*.

## Results

### CASP11, CASP1, and bacterial secretory systems influence GSDMD cleavage in *B. cenocepacia*-infected macrophages

**Figure 1:**
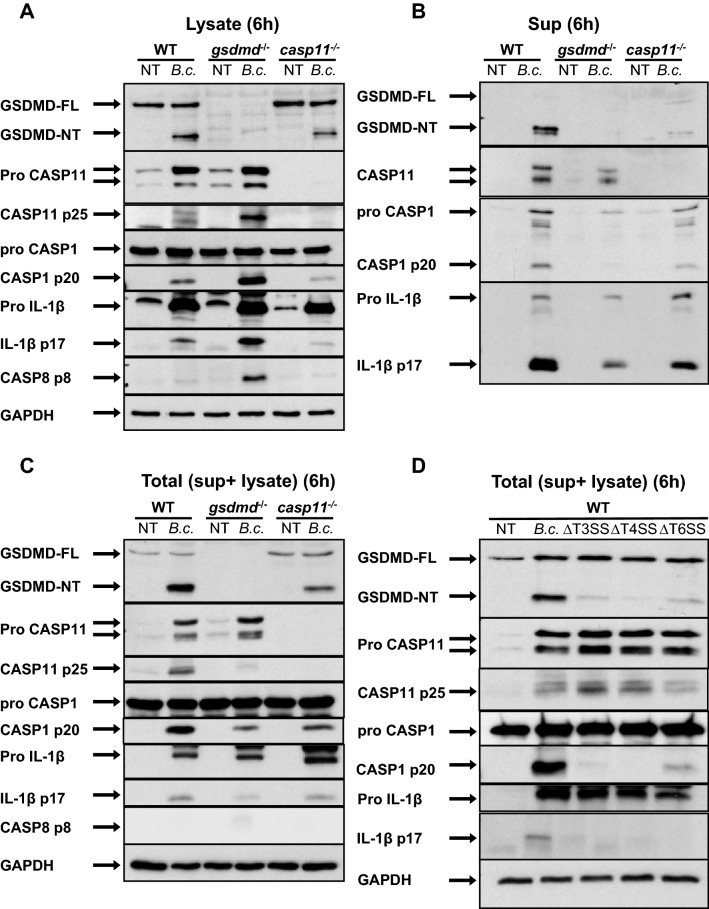
CASP11, CASP1, and bacterial secretory systems influence GSDMD cleavage in *B. cenocepacia*-infected macrophages. **(A)** Immunoblot analysis of GSDMD, CASP11, CASP1, IL-1β, CASP8, and GAPDH in whole cell lysate of WT, *gsdmd*^*−/−*^*,* and *casp11*^*−/−*^ macrophages infected with *B. cenocepacia* (*B.c.*) (MOI10) at 6 h post-infection. **(B)** Immunoblot analysis of GSDMD, CASP11, CASP1, and IL-1β in cell supernatant (sup) from WT, *gsdmd*^*−/−*^ and *casp11*^*−/−*^ macrophages treated as in (**A**). **(C)** Immunoblot analysis of GSDMD, CASP11, CASP1, IL-1β, CASP8, and GAPDH in total cell lysate and sup from WT, *gsdmd*^*−/−*^ , and *casp11*^*−/−*^ macrophages treated as in (**A**). **(D)** Immunoblot analysis of GSDMD, CASP1, CASP11, IL-1β, and GAPDH in total cell lysate and sup from WT macrophages infected with WT *B. cenocepacia*, ∆T3SS, ∆T4SS, and ∆T6SS mutants (MOI10) at 6 h post-infection. (**A**–**D**) Representative blots from 3 independent experiments.

GSDMD-FL is inactive and upon cleavage by CASP1 and CASP11, the active NT cleaved form is released (GSDMD-NT)^10,11^. CASP11 cleaves GSDMD through the non-canonical inflammasome pathway. CASP11 has been shown to restrict Gram-negative bacteria such as *B. cenocepacia, B. pseudomallei, Legionella pneumophila* and *Salmonella *Typhimurium as well as Gram-positive bacteria^12,14–19^. In order to investigate whether GSDMD is cleaved during *B. cenocepacia* infection, bone marrow-derived macrophages from WT, *gsdmd*^*−/−*^, and *casp11*^*−/−*^ mice were infected with *B. cenocepacia* at MOI of 10. At 6 h post-infection, *casp11*^*−/−*^ macrophages displayed decreased cleavage of GSDMD-FL via Western blot in cell lysates, cell culture supernatants and cell lysates combined with supernatants (total) (Fig. 1A–C). These data indicate that CASP11 contributes to GSDMD cleavage during *B. cenocepacia* infection.

Previous studies have shown that the presence of cytosolic lipopolysaccharide (LPS) is responsible for the cleavage and auto-activation of CASP11 ^20,21^. Consequently, the liberation of LPS from the phagosome harboring the pathogens is necessary for CASP11 cleavage^22^. Whether the cleavage of CASP11 post- *B. cenocepacia* infection is influenced by GSDMD has yet to be determined. Notably, we found that the total (cell lysate and supernatants) amount of cleaved CASP11 in response to *B. cenocepacia* is reduced in *gsdmd*^*−/−*^ macrophages compared to WT macrophages (Fig. 1C). It is important to note that due to the absence of GSDMD pores in *gsdmd*^*−/−*^ macrophages, the cleaved form accumulates inside the cell (Fig. 1A). Together, these results indicate that GSDMD contributes to CASP11 cleavage.

In addition to CASP11, GSDMD can also be cleaved by active CASP1 through the canonical inflammasome pathway^15^. CASP1 is active in *casp11*^*−/−*^ and *gsdmd*^*−/−*^ macrophages 6 h post- *B. cenocepacia* infection (Fig. 1A–C). Total amount of cleaved GSDMD is reduced in both *casp11*^*−/−*^ and *casp1*^*−/−*^ macrophages (Figs. 1C, S1A). This data indicate that during *B. cenocepacia* infection, GSDMD is cleaved by both CASP11 (non-canonical inflammasome) and CASP1 (canonical inflammasome).

CASP8 can mediate cell death in the absence of GSDMD^23–25^. To test if this is the case during *B. cenocepacia* infection, we determined whether CASP8 is cleaved at 6 h post- *B. cenocepacia* infection. Notably, cleaved CASP8 accumulated in *gsdmd*^*−/−*^ but not in *casp11*^*−/−*^ cell lysates (Fig. 1A). However, we found that total cleaved CASP8 is barely detected in *gsdmd*^*−/−*^ and *casp11*^*−/−*^ macrophage lysates combined with their supernatants (Fig. 1C).

Bacterial secretory systems (SS) release bacterial virulence factors^26^. These SS are essential for bacterial persistence within host cells. *B. cenocepacia* possesses different types (T) of SS including T3SS, T4SS, and T6SS^27^. The different SS of *B. cenocepacia* play a role in IL-1β activation^13^. To determine which SS contributes to GSDMD cleavage and inflammasome activation, WT macrophages were infected with WT *B. cenocepacia* and different SS mutants for 6 h at MOI of 10. Western blot analysis of the cell lysates combined with the cell culture supernatants (total) demonstrate that infection with *SS*^*−/−*^ mutants led to decreased cleavage of GSDMD, CASP1, and IL-1β (Fig. 1D). *T3SS*^*−/−*^ and *T4SS*^*−/−*^ mutants exerted more cleavage of CASP11 (Fig. 1D). To further test whether different SS play a role in invasion or in the intracellular survival and replication within macrophages, we infected macrophages with the different *B. cenocepacia* mutants for 0.5, and 6 h then determined intracellular colony forming unit (CFU). Despite having fast growth rates in LB (Fig. S1B,C), all the mutants displayed defective replication within macrophages compared to the parental strain (Fig. S1D,E). This is independent of cell death as they cause less or equivalent cell death compared to WT *B. cenocepacia* (Fig. S1F). In contrast to the *T6SS*^*−/−*^ , which shows increased invasion^12^, no difference in invasion between WT and *T3SS*^*−/−*^ or *T4SS*^*−/−*^ could be observed (Fig. S1D). Overall, these results indicate that, both host CASP11 and CASP1 in addition to bacterial T3SS, T4SS, and T6SS contribute to *B. cenocepacia*-induced GSDMD cleavage within macrophages.

### GSDMD contributes to the restriction of *B. cenocepacia* in vitro

**Figure 2:**
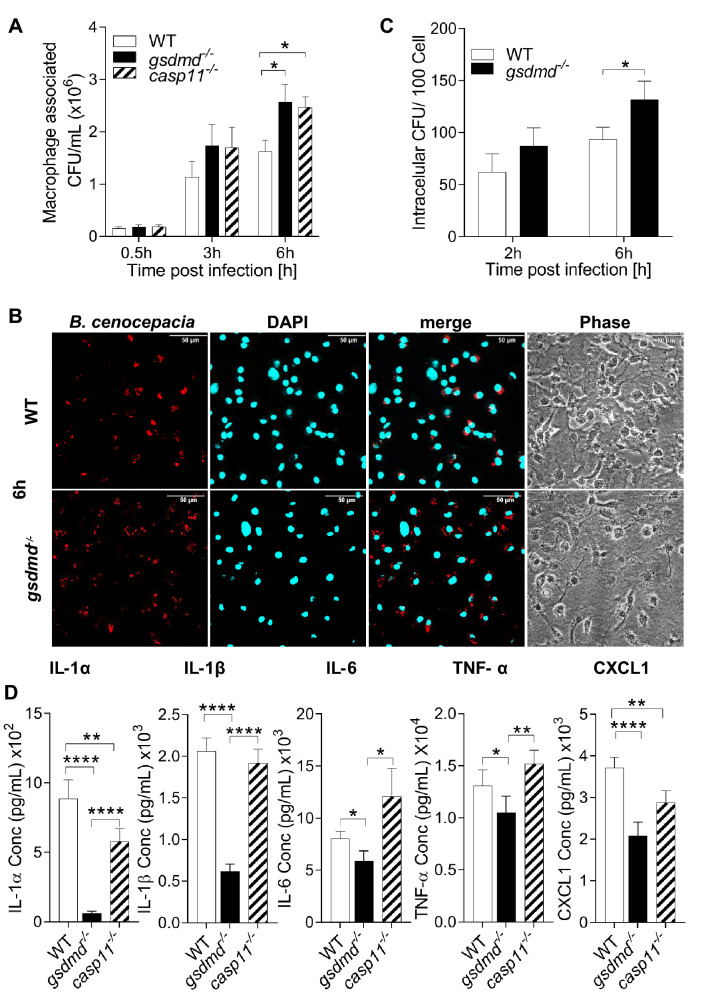
GSDMD contributes to the restriction of *B. cenocepacia *in vitro. **(A)** Macrophage associated colony forming unit (CFU) of *B. cenocepacia* in WT, *gsdmd*^*−/−*^, and *casp11*^*−/−*^ macrophages. Data represent mean ± SEM (n = 10). Statistical analysis was performed using two-way ANOVA. **(B)** Immunofluorescence images of macrophage associated with *B. cenocepacia* in WT and *gsdmd*^*−/−*^ macrophages at 6 h post-infection. Scale bar: 50 µm. **(C)** Quantification of macrophage associated CFU of *B. cenocepacia* in WT and *gsdmd*^*−/−*^ macrophages at 2 and 6 h post-infection (MOI10). Values are mean ± SEM calculated by scoring 20 randomly chosen fields of view from 7 independent experiments and then normalized to the number of cells. Statistical analysis was performed using two-way ANOVA. **(D)** Cytokine release from WT, *gsdmd*^*−/−*^, and *casp11*^*−/−*^ macrophages infected with *B. cenocepacia* (MOI10) at 6 h post-infection. Data represent mean ± SEM (n = 20). Statistical analyses were performed using one-way ANOVA. *p ≤ 0.05, **p ≤ 0.01, ***p ≤ 0.001.

The role of GSDMD during infection differs according to the pathogen. For instance, it enhances *Escherichia coli* replication^28^, while it restricts the replication of *B. thailandensis* and *Legionella pneumophila*^29,30^. However, its distinctive role during *B. cenocepacia* infection is unknown. To unveil this, macrophages from WT, *gsdmd*^*−/−*^ , and *casp11*^*−/−*^ mice were infected with *B. cenocepacia* for 0.5, 3, and 6 h and macrophage associated colony forming units (CFU) were determined. Compared to WT macrophages, both *gsdmd*^*−/−*^ and *casp11*^*−/−*^ macrophages had similar levels of uptake as indicated by similar CFU at 0.5 h, however, they are more permissive to *B. cenocepacia* at 6 h post-infection (Fig. 2A). Additionally, using red fluorescent *B. cenocepacia* (*K56-2*), intracellular bacteria were enumerated after 2 and 6 h (Fig. 2B). *Gsdmd*^*−/−*^ macrophages displayed significantly more *B. cenocepacia* intracellularly when compared to WT at 6 h post-infection (Fig. 2C). These results clearly show that GSDMD can efficiently restrict *B. cenocepacia* infection within macrophages.

We showed previously that *casp11*^*−/−*^ macrophages release less inflammasome-independent cytokines including KC (CXCL1) in their supernatants 6 h post- *B. cenocepacia* infection^12^, yet the mechanism of GSDMD involvement remains unknown. As a pore forming protein, GSDMD may affect the release of IL-1β in addition to inflammasome-independent cytokines. We examined if *gsdmd*^*−/−*^ macrophages differentially release cytokines in comparison to WT and *casp11*^*−/−*^ macrophages post- *B. cenocepacia* infection. Indeed, supernatants from *gsdmd*^*−/−*^ macrophages elicited significantly less IL-1α, IL-1β, IL-6, and TNF-α when compared to WT and *casp11*^*−/−*^ supernatants. Additionally, both *casp11*^*−/−*^ and *gsdmd*^*−/−*^ macrophages had significantly less CXCL1 release in comparison to WT (Fig. 2D). These results demonstrate that GSDMD can restrict *B. cenocepacia* and mediate the release of different inflammasome-dependent and -independent, inflammatory cytokines. Together, in the absence of GSDMD, there is more *B. cenocepacia* yet less cytokine secretion.

### GSDMD restricts *B. cenocepacia* infection irrespective of cell death

**Figure 3:**
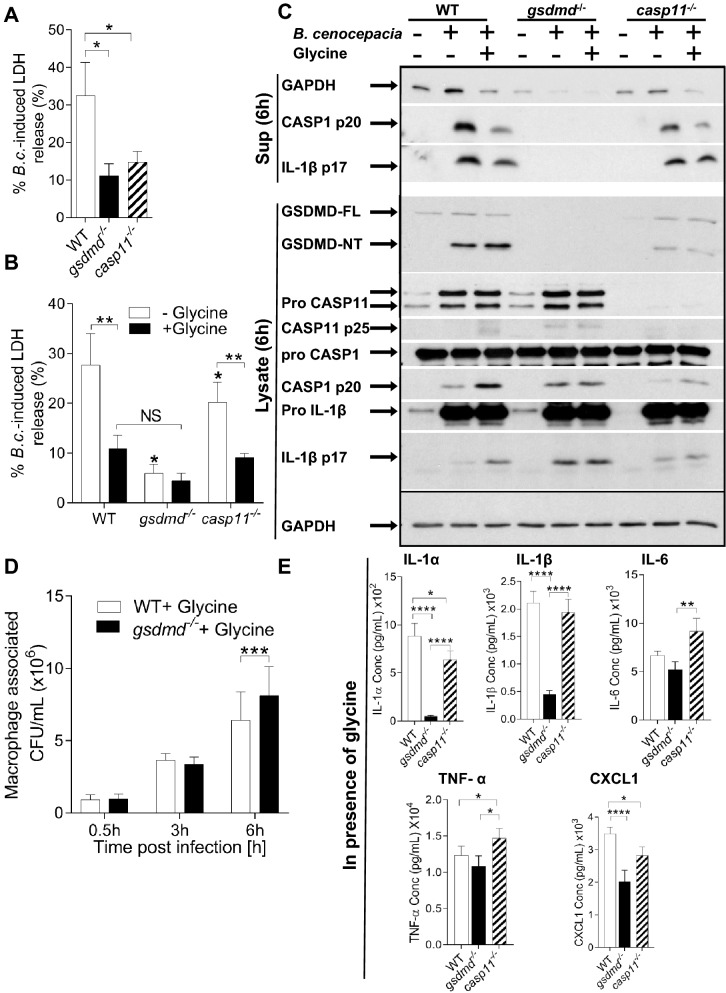
GSDMD restricts *B. cenocepacia* irrespective of cell death. **(A)** *B. cenocepacia* -induced cytotoxicity was calculated by measuring LDH release in supernatants from WT, *gsdmd*^*−/−*^, and *casp11*^*−/−*^ macrophages at 6 h post- *B. cenocepacia* (*B.c.*) infection with (MOI10) (n = 7). Statistical analysis was performed using one-way ANOVA. **(B)** *B. cenocepacia*-induced cytotoxicity was determined as in (**A**) in presence and absence of glycine (5 mM) (n = 5). Statistical analyses were performed using two-way ANOVA. NS = not statistically significant. **(C)** Immunoblot analysis in cell supernatant (Sup) showing GAPDH, CASP1, and IL-1β and in whole cell lysate showing GSDMD, CASP1, CASP11, IL-1β, and GAPDH of WT, *gsdmd*^*−/−*^, and *casp11*^*−/−*^ macrophages infected with *B. cenocepacia* (MOI10) at 6 h post-infection in presence and absence of glycine (5 mM). Representative blots from 3 independent experiments. **(D)** Macrophage associated colony forming unit of *B. cenocepacia* (MOI10) in WT and *gsdmd*^*−/−*^ macrophages at 6 h post-infection in presence of glycine (5 mM). Data represent mean ± SEM (n = 6). Statistical analyses were performed using two-way ANOVA. **(E)** Cytokine release from WT, *gsdmd*^*−/−*^, and *casp11*^*−/−*^ macrophages treated as in (**D**). Data represent mean ± SEM (n = 20). Statistical analyses were performed using two-way ANOVA. *p ≤ 0.05, **p ≤ 0.01, ***p ≤ 0.001.

Pyroptosis is an inflammatory, programmed cell death associated with excess GSDMD pore formation, and characterized by cytokine efflux, water influx, cell swelling, and finally osmotic lysis^31,32^. Cell death can deprive the pathogen from the niche required for its persistence and growth. To investigate if GSDMD-mediated restriction of *B. cenocepacia* is due to its function as an executer of pyroptotic cell death, the release of lactate dehydrogenase (LDH) was measured in supernatants of WT, *gsdmd*^*−/−*^ , and *casp11*^*−/−*^ macrophages at 6 h post- *B. cenocepacia* infection. G*sdmd*^*−/−*^ and *casp11*^*−/−*^ macrophages exhibited lower levels of LDH released into supernatants when compared to WT (Fig. 3A), indicating that WT macrophage undergo more pyroptotic cell death compared to *gsdmd*^*−/−*^ and *casp11*^*−/−*^ macrophages. To examine if increased cell death in WT macrophages is responsible for *B. cenocepacia* restriction and not through other potential functions of GSDMD, glycine was used to protect WT macrophages from cell lysis. Glycine has cytoprotective properties against pyroptosis as it buffers the extracellular media and prevents ion influx, thereby inhibiting swelling and cellular lysis^33–37^. When treated with glycine, *B. cenocepacia* growth or gentamicin sensitivity in LB were not affected (Fig. S2A). WT, *gsdmd*^*−/−*^ , and *casp11*^*−/−*^ macrophages were infected for 6 h in the absence or presence of glycine (5 mM), and the release of LDH and GAPDH were determined. Infected WT macrophages, in the presence of glycine, displayed significantly lower levels of LDH and GAPDH release compared to those not treated with glycine. Thus, in the presence of glycine, there was no significant difference in the LDH and GAPDH release among WT, *gsdmd*^*−/−*^ and *casp11*^*−/−*^ macrophages (Fig. 3B,C). To determine if protection against pyroptosis affects the production and secretion of inflammatory proteins, WT, *gsdmd*^*−/−*^ and *casp11*^*−/−*^ macrophages were infected with *B. cenocepacia* in the absence or presence of glycine. Compared to the non-glycine treated macrophages, WT macrophages displayed decreased CASP1 and IL-1β in the cell supernatant. Conversely, they exhibited more expression of cleaved CASP11, CASP1 and IL-1β in cell lysate in the presence of glycine (Fig. 3C). These results indicated that glycine pre-treatment of macrophages can efficiently protect against pyroptosis during *B. cenocepacia* infection.

We then determined if *gsdmd*^*−/−*^ macrophages would still be more permissive to *B. cenocepacia* than WT cells during glycine treatment. Macrophages from WT and *gsdmd*^*−/−*^ mice were infected with *B. cenocepacia* and CFU were determined at 0.5, 3, and 6 h in the presence of glycine. Both WT and *gsdmd*^*−/−*^ macrophages exhibited similar invasion of *B. cenocepacia.* However, *gsdmd*^*−/−*^ macrophages were more permissive at 6 h post-infection compared to glycine-protected WT cells (Fig. 3D). These results confirm that GSDMD plays a role in hindering *B. cenocepacia* replication in addition to its function in mediating cell death.

WT macrophages release more cytokines than *gsdmd*^*−/−*^ macrophages (Fig. 2D), however, WT cells exhibited higher levels of cell death during *B. cenocepacia* infection which may lead to increased inflammatory cytokine release (Fig. 3A). Therefore, we sought to decipher the contribution of GSDMD pore formation or cell lysis to inflammatory cytokine release. To answer this question, we added glycine, which does not block GSDMD pores but does protect against lytic cell death, and collected supernatant 6 h post- *B. cenocepacia* infection^38^. Glycine treatment and the prevention of cell lysis reduced the amount of IL-1α, IL-1β, and IL-6 in *gsdmd*^*−/−*^ supernatants (Fig. S2B). Glycine-treated *gsdmd*^*−/−*^ macrophage supernatants still had significantly less IL-1α, IL-1β, and CXCL1 in comparison to supernatants of WT macrophages, but not TNF-α or IL-6, indicating that the release of these cytokines was not mainly dependent on cell lysis (Fig. 3E). In addition, WT cells exhibited similar amounts of IL-1α, IL-1β, and TNF-α release with and without treatment, but lower levels of CXCL1 and IL-6 in infection supernatants (Fig. S2B). Together, these results confirm that GSDMD promotion of IL-1α, IL-1β, and CXCL1 release is not dependent on cell lysis in vitro.

### GSDMD restricts the replication and dissemination of *B. cenocepacia* in vivo

**Figure 4:**
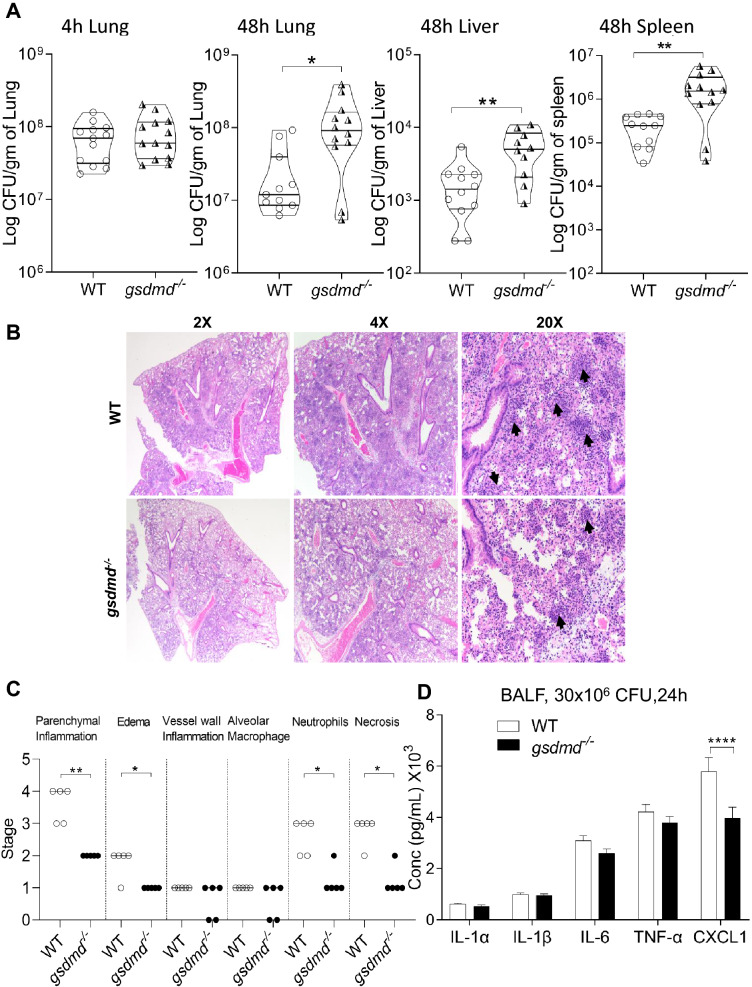
GSDMD restricts the replication and dissemination of *B. cenocepacia *in vivo. **(A)** In vivo CFU from lung, liver, and spleen of WT and *gsdmd*^*−/−*^ mice infected with *B. cenocepacia* (10 × 10^6^ CFU/mouse) at 4 h (n = 10) and 48 h (n = 13). Pooled data from 3 independent experiments are shown as mean ± SEM. Statistical analysis was performed using unpaired two-tailed student’s t-test. **(B)** Representative 2x (left panel), 4x (middle panel), 20x (right panel) magnification of H&E-stained lung sections from WT (upper panel) and *gsdmd*^*−/−*^ (lower panel) mice (n = 5) treated as in (**A**) for 48 h. Black arrows show the inflammatory cells infiltration. **(C)** Scatter dot blot showing the difference in the histological stages (parenchymal inflammation, perivascular and peribronchiolar edema, inflammatory cells in blood vessel walls, alveolar macrophages, percentage of affected lung containing neutrophils/neutrophil clusters, percentage of affected lung composed of necrosis/necrotic debris) between lung sections from WT and *gsdmd*^*−/−*^ mice (n = 5) treated as in (**A**) for 48 h. Statistical analysis was performed using Mann–whitney test. 0: Absent; 1: mild; 2: moderate; 3: marked; 4: severe; 5: fully. **(D)** Cytokine levels at 24 h in the bronchoalveolar lavage fluid (BALF) of WT and *gsdmd*^*−/−*^ (n = 20) mice after intratracheally infection with 30 × 10^6^ CFU/mouse. Statistical analysis was performed using two-way ANOVA. *p ≤ 0.05, **p ≤ 0.01, ***p ≤ 0.001.

To test if GSDMD plays a role in *B. cenocepacia* replication in vivo, WT and *gsdmd*^*−/−*^ mice were infected intratracheally with a sub-lethal dose (10 × 10^6^ CFU/mouse) of *B. cenocepacia*. To verify if equal inoculums of bacteria were delivered to all mice, CFU enumeration was performed on the lung homogenates 4 h post-infection. There was no significant difference in the bacterial loads between WT and *gsdmd*^*−/−*^ mice (Fig. 4A). However, after 48 h the bacterial burden was significantly increased in the lung, spleen, and liver of *gsdmd*^*−/−*^ mice when compared to the corresponding WT mice (Fig. 4A). This indicates that GSDMD promotes the restriction of *B. cenocepacia* replication and prevents dissemination in vivo. To determine if the permissiveness of *gsdmd*^*−/−*^ is associated with mortality, WT and *gsdmd*^*−/−*^ mice were monitored after infection intratracheally with lethal and sub-lethal doses. Interestingly, no significant difference in the survival rate was detected between WT and *gsdmd*^*−/−*^ mice in all the indicated doses (Fig. S3A–C), indicating that higher bacterial burden in the absence of GSDMD is not associated with increased mortality.

To test if GSDMD plays a role in promoting inflammation in vivo, lung tissues were collected from WT and *gsdmd*^*−/−*^ mice infected with 10 × 10^6^ CFU/mouse for 48 h. Hemotoxylin and Eosin-staining was performed to assess morphological and histological changes. Overall, infection of WT lungs was accompanied with severe inflammatory infiltrates and a larger percentage of the lung tissue inflammation when compared to *gsdmd*^*−/−*^ lungs (Fig. 4B). Semi-quantitative histopathological scoring demonstrates that the infected WT lung tissues have significantly more areas of inflammation, edema, and necrosis than their *gsdmd*^*−/−*^ counterparts. Additionally, they exhibited greater neutrophil infiltration, and clusters mixed with necrotic debris in the parenchyma, affecting alveoli, capillaries and small blood vessels (Fig. 4B,C). Myeloperoxidase (MPO) is one of the degrading enzymes typically produced by neutrophils^39,40^. To determine if decreased neutrophil infiltration, in the absence of GSDMD, correlates with decreased cytokine and/or MPO release, we collected the bronchoalveolar lavage fluid (BALF) from WT and *gsdmd*^*−/−*^ mice infected intratracheally with *B. cenocepacia* (30 × 10^6^ CFU/mouse) for 24 h and measured various cytokines IL-1α, IL-1β, IL-6, TNF-α, CXCL1 in addition to MPO. There was no significant difference in all the measured cytokines in addition to MPO, except for CXCL1 which was less in *gsdmd*^*−/−*^ BALF (Fig. 4D, S3D). Overall, these results show that GSDMD contributes to the restriction of *B. cenocepacia* replication, lung inflammation, and BALF CXCL1 production in vivo. However, the mechanism of GSDMD-mediated restriction of *B. cenocepacia* is still unclear.

### GSDMD contributes to bactericidal mROS production during *B. cenocepacia* infection

**Figure 5:**
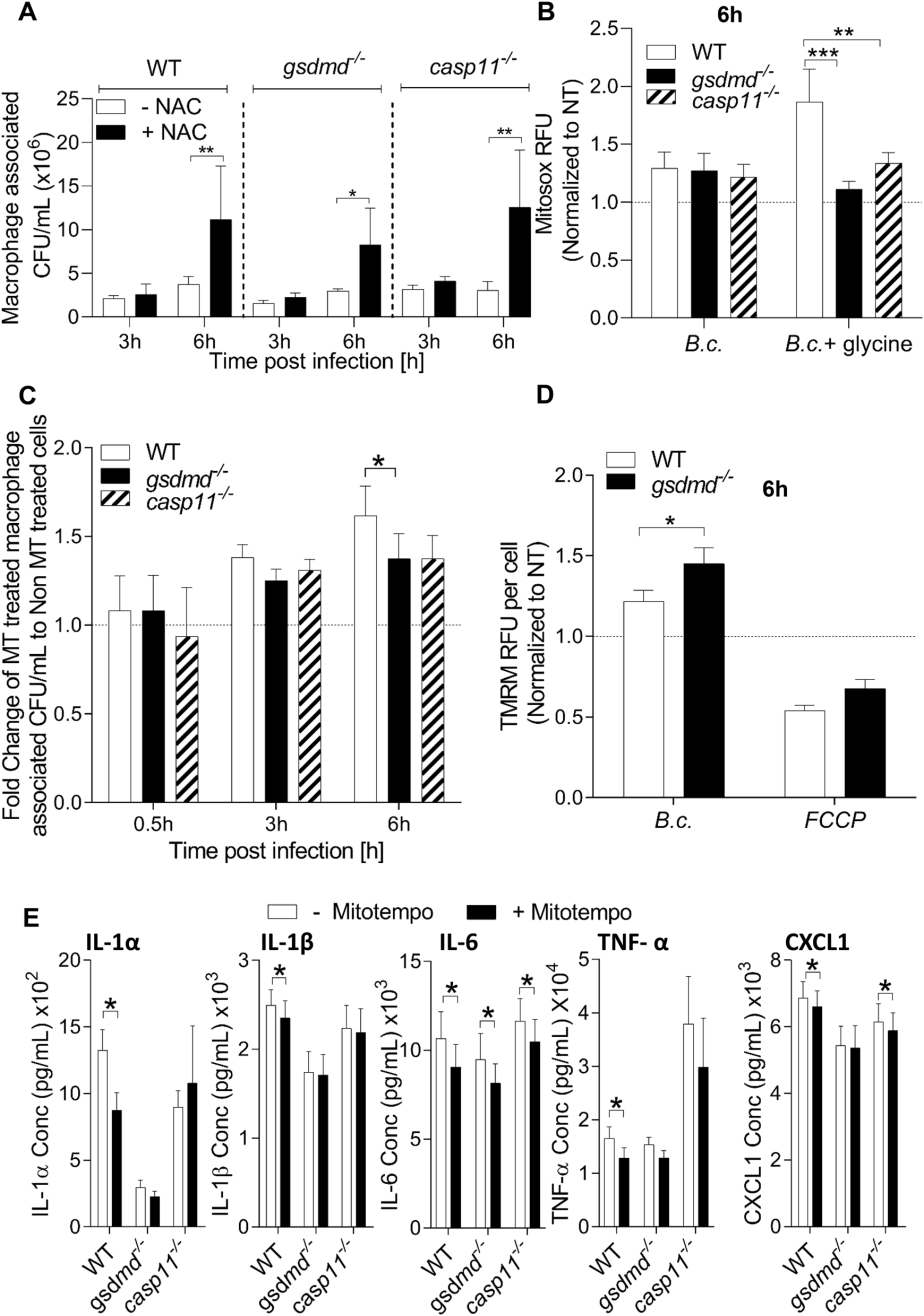

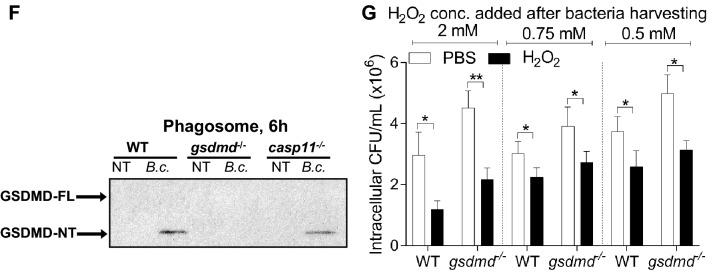
GSDMD contributes to bactericidal mROS production during *B. cenocepacia* infection. **(A)** Macrophage associated CFU of *B. cenocepacia* (*B.c.)* (MOI10) in WT, *gsdmd*^*−/−*^, and *casp11*^*−/−*^ macrophages in presence and absence of N-acetylcysteine (NAC) (3 mM). Data represent mean ± SEM (n = 3). Statistical analysis was performed using two-way ANOVA. **(B)** Mitosox assay in WT, *gsdmd*^*−/−*^*, and casp11*^*−/−*^ macrophages infected with *B. cenocepacia* (*B.c.*) (MOI10) at 6 h post-infection in absence or presence of glycine (5 mM). Data represent mean ± SEM (n = 8). Statistical analysis was performed using two-way ANOVA. **(C)** Fold change comparing the addition of Mitotempo (MT) (20 µM) treatment in macrophage associated CFU of *B. cenocepacia* (MOI10) in WT, *gsdmd*^*−/−*^, and *casp11*^*−/−*^ macrophages at 6 h infection. Data represent mean ± SEM (n = 10). Statistical analysis was performed using two-way ANOVA. **(D)** TMRM assay in WT and *gsdmd*^*−/−*^ macrophages infected with *B. cenocepacia* (MOI10) at 6 h post-infection. FCCP (uncoupler cause mitochondrial membrane potential (MMP) dissipation) (10 µM) was included as negative control. Fluorescence values were normalized to cell number as measured per well before normalization to the fluorescence of the non-infected wells. Data represent mean ± SEM (n = 12). Statistical analysis was performed using two-way ANOVA. **(E)** Cytokine release from WT, *gsdmd*^*−/−*^*,* and *casp11*^*−/−*^ macrophages treated as in (**C**). Data represent mean ± SEM (n = 11). Statistical analysis was performed using paired one-tailed student’s t-test. **(F)** Immunoblot analysis of GSDMD in isolated phagosomes from WT, *gsdmd*^*−/−*^, and *casp11*^*−/−*^ macrophages infected with *B. cenocepacia* (MOI10) at 6 h post-infection. **(G)** CFU of *B. cenocepacia* harvested from WT and *gsdmd*^*−/−*^ macrophages and treated with H_2_O_2_ for 30 min before plating. Data represent mean ± SEM (n = 3). Statistical analysis was performed using two-way ANOVA. *p ≤ 0.05, **p ≤ 0.01, ***p ≤ 0.001.

One of the main challenges to the intracellular lifestyle of *B. cenocepacia* is the production of host reactive oxygen species (ROS)^41–44^, however it is unclear if GSDMD contributes to the production of mROS during *B. cenocepacia* infection and if it contributes to the restriction of the organism. Therefore, we used the antioxidant N-acetylcysteine (NAC) to determine if decreasing ROS will allow more *B. cenocepacia* replication within macrophages. Since NAC (3 mM) was found to have a direct effect on *B. cenocepacia* replication in LB (Fig. S4A)*,* it was added to macrophages post. *B. cenocepacia* infection. Despite increasing cell death (Fig. S4B), scavenging ROS significantly increased *B. cenocepacia* replication in all the infected genotypes (Fig. 5A). This indicates that ROS manipulation can significantly affect *B. cenocepacia* survival within macrophages.

GSDMD inserts into mitochondrial membranes, when activated, contributing to mitochondrial ROS (mROS) release which is the major source of ROS in the cell ^45^. Thus, we postulated that *gsdmd*^*−/−*^ macrophages produce less mROS during *B. cenocepacia* infection, contributing to *B. cenocepacia* permissiveness. We treated WT and *gsdmd*^*−/−*^ macrophages with MitoSOX dye which selectively targets the mitochondria where it is oxidized, producing a highly fluorescent product that is used for mROS measurement. We found that WT and *gsdmd*^*−/−*^ macrophages induced comparable mROS during *B. cenocepacia* infection (Fig. 5B). Yet, the difference in mROS production between the macrophage genotypes could be masked by earlier death of WT macrophages. Therefore, glycine was added and the amount of mROS was determined. Notably, *gsdmd*^*−/−*^ and *casp11*^*−/−*^ macrophages produced lower mROS in the presence of glycine when compared to non-pyroptotic WT cells (Figs. 5B, S4C). To further confirm if the defective mROS production associated with *gsdmd*^*−/−*^ macrophages contributes to permissiveness to *B. cenocepacia*, Mitotempo (MT), a selective mitochondria-targeted antioxidant, was used^46^. MT does not induce macrophage death (Fig. S4D) and has no direct bactericidal effect against *B. cenocepacia* (Fig. S4E). WT, *gsdmd*^*−/−*^, and *casp11*^*−/−*^ macrophages were infected with *B. cenocepacia* for 0.5, 3, and 6 h in the absence and presence of MT (20 µM) then, macrophage associated bacterial CFUs were enumerated. *B. cenocepacia* replication increased in all the infected macrophage phenotypes, however this increase was more significant in MT-treated WT macrophages compared to *gsdmd*^*−/−*^ macrophages at 6 h post-infection (Fig. 5C). Together, these results demonstrate that mROS contributes to the restriction of *B. cenocepacia* and that WT macrophages produce more mROS than *gsdmd*^*−/−*^ and *casp11*^*−/−*^ macrophages during *B. cenocepacia* infection however, the mechanism is unclear.

GSDMD inserts pores in mitochondrial membranes which can increase the production of mROS by disrupting mitochondrial membrane potential (MMP) (ΔΨ_m_)^45,47–52^. To determine if GSDMD disrupts MMP (ΔΨ_m_) during *B. cenocepacia*. TMRM dye (10 nM) was added 0.5 h before the designated time point (6 h post- *B. cenocepacia* infection). This dye accumulates in a negatively charged polarized mitochondria giving orange fluorescence. On the other hand, when MMP is disrupted, the dye is dispersed throughout the cell cytosol and fluorescence levels drop dramatically. Accordingly, MMP was found to be higher in *gsdmd*^*−/−*^ macrophages during *B. cenocepacia* infection in comparison to WT counterparts (Fig. 5D). This indicates that activation and cleavage of GSDMD during *B. cenocepacia* infection leads to disruption of MMP.

Previous studies demonstrated that in human peripheral blood mononuclear cells, mROS promotes production of pro-inflammatory cytokines^53–55^. This led us to explore if defective mROS production in *gsdmd*^*−/−*^ macrophages during *B. cenocepacia* infection contributes to lower cytokine levels (Fig. 3E). By inhibiting mROS via MT, we detected significantly lower pro-inflammatory cytokines release (IL-1α, IL-1β, CXCL1, IL-6 and TNF-α) in the cell culture supernatants of WT murine macrophages 6 h post- *B. cenocepacia* infection (Fig. 5E). In addition, there was no significant difference in the release of cytokines, except for IL-6, in *gsdmd*^*−/−*^ macrophages upon treatment with MT. These data indicate that GSDMD-induced mROS contribute to the secretion of pro-inflammatory cytokines from murine macrophages during *B. cenocepacia* infection.

### GSDMD is recruited to the *B. cenocepacia*-containing phagosome but does not alter the integrity of the* bacterial* membrane

To determine if GSDMD co-localizes with *B. cenocepacia*, we purified phagosomes from *B. cenocepacia*-infected WT macrophages. We then used Western blot analysis to identify the presence of the cleaved GSDMD on the isolated phagosomes from WT macrophages infected with *B. cenocepacia* for 6 h (Fig. 5F). We found that cleaved but not full-length GSDMD is present on the phagosomal compartment of the *B. cenocepacia*-infected WT macrophages^56,57^. GSDMD was found to have direct bactericidal effect on the membranes of *B. thailandensis,* by attacking their membrane, thereby making them more vulnerable to H_2_O_2_ (2 mM)^30,50,58^. To test if the presence of GSDMD on the phagosomes containing *B. cenocepacia* renders the bacterium more susceptible to H_2_O_2_, WT and *gsdmd*^*−/−*^ macrophages were infected with *B. cenocepacia* for 6 h in the presence of glycine before treating the harvested intracellular bacteria with different concentration of H_2_O_2_ for 30 min (2, 0.75, 0.5 mM), and the number of the surviving bacteria was determined (Fig. 5G). H_2_O_2_ significantly decreased the number of bacteria harvested from both WT and *gsdmd*^*−/−*^ macrophages, indicating that there is no difference in the H_2_O_2_ susceptibility of intracellular *B. cenocepacia* in the presence of GSDMD. Therefore, we concluded that despite its presence on the phagosomes harboring *B. cenocepacia*, GSDMD does not have a direct bactericidal effect against *B. cenocepacia* within macrophages.

### *Gsdmd*^*−/−*^ mice and their derived macrophages exhibit less autophagosome formation during *B. cenocepacia* infection

Autophagy is a conserved pathway among eukaryotic cells that degrades cytosolic non-functional organelles, protein aggregates, phagocytosed particles, and intracellular pathogens. It plays an important role in *B. cenocepacia* clearance from macrophages^59^. Several studies suggested that GSDMD contributes to the release of mROS which mediates the stimulation of autophagy and bacterial clearance^60–62^. To test this association, WT macrophages were treated with the selective mROS inducer (MitoPQ) and the mROS inhibitor (MT) for 4 h. Autophagy flux was accessed using Bafilomycin A1 (BafA1) which was added 2 h before the designated time point^17,63,64^. Then, the detection of the cleavage and lipidation of LC3-I into LC3-II was determined. Compared to the non-treated cells, MT treatment led to loss of the autophagic flux in WT macrophages. Accordingly, increasing mROS production by MitoPQ was accompanied by increase of the autophagy flux (Fig. S5A,B). These results demonstrate that mROS positively regulate autophagy in macrophages.

**Figure 6:**
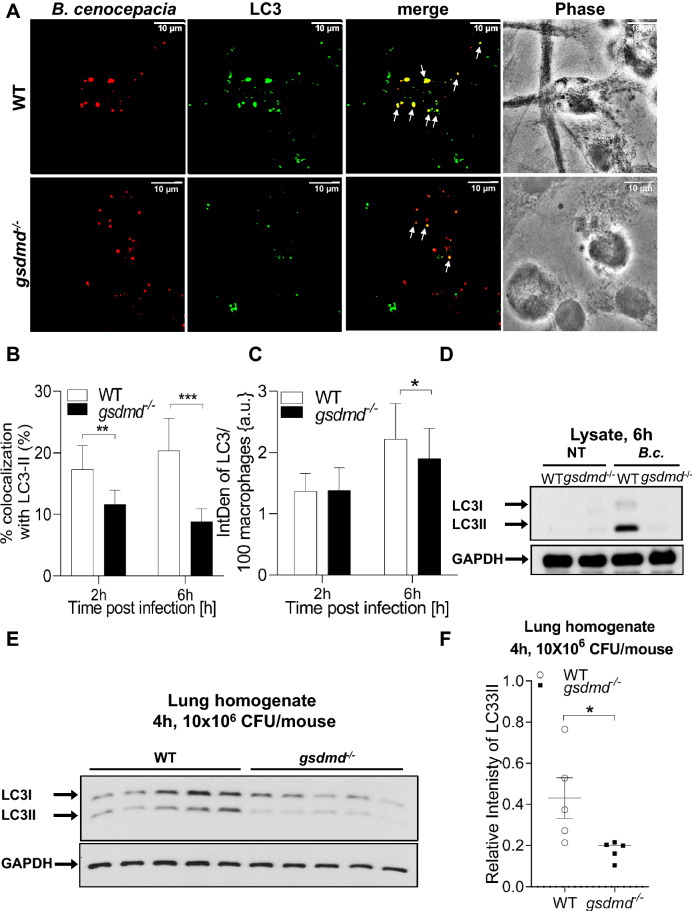
Gsdmd^*−/−*^ mice and their macrophages exhibit less autophagosme formation during *B. cenocepacia* infection. (**A**) Confocal images of LC3 (green) immunofluorescence assay of *B. cenocepacia* (*B.c.*) (red) (MOI10) infected WT and *gsdmd*^*−/−*^ macrophages at 6 h post-infection. White arrows points to *B. cenocepacia*- LC3 co-localization. Scale bar: 10 µM. (**B**) Quantification of *B. cenocepacia* co-localized with LC3 in *B. cenocepacia* infected WT *and gsdmd*^*−/−*^ macrophages treated as in (**A**). Data represent mean ± SEM (n = 5). Statistical analysis was performed using two-way ANOVA. (**C**) Quantification of total LC3 puncta in *B. cenocepacia* infected WT *and gsdmd*^*−/−*^ macrophages treated as in (**A**) using ImageJ Software. Values are mean percentage ± SEM calculated by scoring 96 randomly chosen fields of view from 6 independent experiments. Statistical analysis was performed using paired two tailed Student’s t-test.(**D**) Immunoblot analysis of LC3 in whole cell lysate separated of WT and *gsdmd*^*−/−*^ macrophages treated as in (**A**). (**E**) Immunoblot analysis of LC3 in lung homogenate separated of WT and *gsdmd*^*−/−*^ mice after being infected intratracheally for 4 h by *B. cenocepacia* (10 × 10^6^ CFU/mouse) (n = 5). (**F**) Densitometry analysis of immunoblots represented in (**E**). Data represent mean ± SEM (n = 5). Statistical analysis was performed using unpaired two-tailed student’s t-test *p ≤ 0.05, **p ≤ 0.01, ***p ≤ 0.001.

On the other hand, we and others have demonstrated that *B. cenocepacia* inhibits autophagy regulatory gene expression^59,65^. Additionally, the reduced activation and release of the pro-inflammatory cytokines negatively regulate autophagy^55,66^. In *gsdmd*^*−/−*^ macrophages, there is higher *B. cenocepacia* loads along with defective mROS production in addition to defective cytokine release. Together, this led us to hypothesize that these macrophages elicit defective autophagy, which in turn reduces *B. cenocepacia* clearance. Functional autophagy is depicted by the formation of autophagosomes, called puncta, which can be monitored using confocal microscopy^12^. To test our hypothesis, WT and *gsdmd*^*−/−*^ macrophages were infected with *B. cenocepacia* and autophagosomes were tracked using an antibody against the autophagy marker LC3. Remarkably, *gsdmd*^*−/−*^ macrophages had less *B. cenocepacia* co-localization with LC3 at 2, and 6 h post-infection compared to WT cells (Fig. 6A,B). Additionally, GSDMD deficiency led to significantly less LC3 puncta formation at 6 h post-infection (Fig. 6C). Furthermore, we quantified the LC3-II level in response to *B. cenocepacia* via western blot. Compared to corresponding WT macrophages, LC3-II was significantly reduced within *gsdmd*^*−/−*^ macrophages (Fig. 6D, S5C). Therefore, GSDMD is required for autophagosome formation during *B. cenocepacia* infection in vitro.

**Figure 7:**
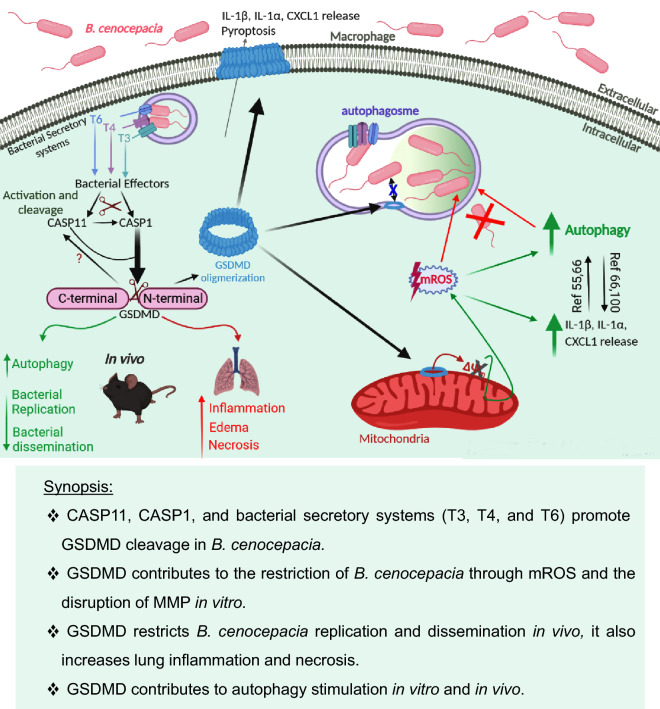
Schematic illustration of the interplay between GSDMD, mROS, autophagy, cytokines release and *B. cenocepacia* restriction. T: type, CASP11: Caspase11, CASP1: Caspase1, GSDMD: Gasdermin D, IL-1β: Interleukin-1β, IL-1α: Interleukin-1α, CXCL1: C-X-C Motif Chemokine Ligand 1, mROS: mitochondrial reactive oxygen species, ΔΨm: mitochondrial membrane potential, Ref: references.

To determine if it plays a similar role in vivo, the lungs of WT and *gsdmd*^*−/−*^ mice, infected intratracheally with *B. cenocepacia* (10 × 10^6^ CFU/mouse) for 4 h, were homogenized and analyzed to determine the amount of LC3II*.* Compared to corresponding WT lungs, LC3-II was significantly reduced within *gsdmd*^*−/−*^ lungs (Fig. 6E,F). Together these data show that during *B. cenocepacia* infection, autophagy is compromised in the absence of GSDMD both in vitro and in vivo. A schematic illustration of the interplay between GSDMD, mROS, autophagy, cytokines release and *B. cenocepacia* replication is suggested in (Fig. 7).

## Discussion

*B. cenocepacia* is a classic opportunistic pathogen characterized by an inherent multidrug resistance^67^. To this end, better understanding of host–pathogen interaction and the discovery of new host-based approaches that can help control this opportunistic pathogen are clinically exigent. Our latest findings have characterized the crucial role of CASP11 in the restriction of *B. cenocepacia*^12^. CASP11 and/or CASP1 can cleave and activate GSDMD^11^, yet, its role in *B. cenocepacia* infection is not defined. In this study, we identify the requirements for GSDMD activation in response to *B. cenocepacia* and the downstream effect on *B. cenocepacia* growth in vivo and in vitro.

GSDMD inhibits the replication of different bacteria including *B. thailandensis*^19,29,30^, but this is not the case for all bacteria. Particularly, GSDMD enhances the replication of other bacteria such as *Escherichia coli*^28^. As a pore forming protein that targets cardiolipin, GSDMD inserts into the mitochondrial membrane and disturbs the mitochondrial membrane potential, leading to mROS production during *B. cenocepacia* infection as reported for other microbes^45,68^. At present, several prominent reports demonstrated that mitochondria produce less mROS at high membrane potential which corroborates our findings with *B. cenocepacia*^45,47–52^. Subsequently, mROS can be delivered to bacteria-harboring phagosomes via mitochondrial vesicles contributing to bacterial clearance^69,70^. The bactericidal effect of mROS against different Gram-negative and Gram-positive bacteria including *Salmonella *Typhimurium^71^ and *Staphylococcus aureus*^70^, has been described, nevertheless, the contribution of GSDMD was not examined.

On the other hand, mROS were shown to stimulate autophagy, which is an important macrophage defense mechanism against intracellular bacterial pathogens including *B. cenocepacia*^59,65,72^. The activation of autophagy promotes the clearance of *B. cenocepacia*^12^, *Legionella pneumophila*^18^*, Listeria monocytogenes*^73^*, and Mycobacterium tuberculosis*^74^*.* Increased mROS production which activates autophagy improves the clearance of *Mycobacterium tuberculosis*^75^, *Streptococcus pneumoniae*^76^ and *Listeria monocytogenes*^77^. Here, we found that autophagy is defective in *gsdmd*^*−/−*^ mice and their derived macrophages. Notably, increasing mROS production in macrophages stimulates autophagy and improved *B. cenocepacia* clearance. Remarkably, phagosomes isolated from *B. cenocepacia*-infected macrophages harbored cleaved GSDMD within their membranes. It is possible that the formation of GSDMD pore on phagosomal membranes recruits autophagy in an attempt to repair the pore as it happens at the cell membrane^78^. This will promote uptake by autophagosomes leading to bacterial degradation. Another possibility is that GSDMD pores contribute to the efflux of potassium K ^+^ ^79^ which promotes the activation of autophagy^80^. It is also possible that GSDMD pore, at the plasma membrane, mediates Ca^2 +^ influx^81^ which may alter the activation of autophagy^82^. Therefore, the mechanisms by which GSDMD activation may promote autophagy activity are numerous.

On the other hand, GSDMD can exert direct bactericidal effect against the membranes of few Gram-negative bacteria within macrophages making them more susceptible to the effect of H_2_O_2_. These include *B. thailandensis*^30^, *Salmonella *Typhimurium^58^ and *Escherichia coli*^50^. Yet, *B. cenocepacia* harvested from WT macrophages were not more susceptible to H_2_O_2_ when compared to those obtained from *gsdmd*^*−/−*^ macrophages. This could be attributed to either the presence of GSDMD on the phagosomal membrane without directly attacking the bacteria within this phagosomes as Liu *et al.* proposed^50^ and/or due to the thick polysaccharide on the membrane of *B. cenocepacia* as Byluand *et* *al.* suggested^83^ for other organisms*.*

Although *gsdmd*^*−/−*^ mice exhibited increased bacterial load in different organs in our study, there was no significant difference in their survival compared to the WT control. Recent reports demonstrated that GSDMD similarly restricted bacteria, but in contrast to our study, GSDMD promoted survival of mice infected with *B. thailandensis* and *Francisella novicida*^30,84,85^. Although, increased bacterial loads are typically associated with decreased survival in mice^12,86–88^, this association is not consistent with inflammation^89,90^. As such, GSDMD can play a conflicting role during in vivo infections by promoting cytokine production, immune cell recruitment and tissue damage while restricting bacterial infection^28^. This explains why the absence of GSDMD does not alter the survival of the mice in response to elevated *B. cenocepacia* loads*.*

In agreement with this hypothesis, we found that WT lungs exhibited, histologically, more inflammation, neutrophils clustering, and tissue necrosis in comparison to *gsdmd*^*−/−*^ lungs. This is consistent with what we found before in *casp11*^*−/−*^ lungs in response to *B. cenocepacia*^12^. This can be attributed to the difference in cytokine release, especially the release of the pro-inflammatory cytokines which mediate recruitment of immune cells^91^. The role of CASP11 in the release of pro-inflammatory cytokines and chemokines in bronchoalveolar lavages (BALFs) varies upon infection with different bacterial pathogens. For instance, CASP11 deficiency during *B. cenocepacia* infection leads to less IL-1β, IL-1α, and CXCL1 production^12^. On the other hand, in *B*. *thailandensis* and *Acinetobacter baumannii* infection, IL-1β ' secretion in BALF is greatly affected by the lack of CASP11^30,92^. In a similar fashion, BALF from *gsdmd*^*−/−*^ mice contains less IL-18 in response to *B*. *thailandensis*^30^. Here we found that, with the exception of CXCL1, which promotes neutrophil trafficking, IL-1β, IL-1α, IL-6, and TNFα were equivalently released in the lungs of the different mice strains which infected with *B. cenocepacia*^93,94^. Hence, it is plausible to conclude that the release of CXCL1 in the BALF during *B. cenocepacia* infection is GSDMD dependent, and promotes inflammation within the lungs. In vitro, GSDMD controls the release of not only the inflammasome-dependent cytokines (IL-1β and IL-1α) but also the independent one (CXCL1) in response to *B. cenocepacia* infection. We used glycine to rule out the possibility that cell death contributes to the increase release of cytokines in specific strains. This is in accordance with other studies, which used glycine as a cytoprotective agent, during *Candida albicans*^36,95^, *Salmonella* Typhimurium^34,37^, and *B. thailandensis*^30^ infections. Intracellularly, the lysate of *gsdmd*^*−/−*^ macrophages exhibited an accumulation of mature IL-1β. This can be explained by the absence of GSDMD pores and CASP8 activation, which can also cleave proIL-1β^96,97^. Notably, we found increased CASP8 activation in response to *B. cenocepacia* infection in *gsdmd*^*−/−*^ macrophages as reported in previous studies^23–25,98^. The intracellular accumulation of mature IL-1β in *gsdmd*^*−/−*^ macrophages can lead to feedback inhibition of CASP1 cleavage and IL1β processing in response to *B. cenocepacia*. The lower occurrence of their cleaved forms in the total supernatant and cell lysate of *gsdmd*^*−/−*^ macrophages, despite the increased bacterial load, lend weight to this hypothesis of a negative feedback loop, however, it requires further validation. Besides IL-1β, we found less IL-1α in the supernatants of *gsdmd*^*−/−*^ macrophages indicating that GSDMD pore also plays a role in IL-1α release. GSDMD activation in response to *B. cenocepacia* infection may increase CASP11 expression and cleavage by the released IL-1α, IL-1β as we previously reported^99^. Furthermore, the cytokines released from WT macrophages (IL-1α, IL-1β, and CXCL1) were found to be decreased in WT supernatants after reducing mROS production in response to *B. cenocepacia*. This emphasizes the role of mROS in bolstering cytokine production^53–55^. Consequently, the aforementioned lower secretion of IL-1α, IL-1β, and CXCL1 in *gsdmd*^*−/−*^ macrophages could be attributed to the defective mROS in these cells during *B. cenocepacia* infection. Additionally, defected autophagy in *gsdmd*^*−/−*^ macrophages may contribute in the decreased secretion of cytokines to the extracellular milieu as it has been proposed before^100^.

It is worth noting that GSDMD cleavage could be downstream of ROS production during canonical inflammasome activation^101^. On the other hand, in non-canonical inflammasome activation, mROS are believed to be downstream of GSDMD which provides a link by which the non-canonical pathway may activate the canonical inflammasome^45^. The decreased expression of CASP1 together with defective mROS production in *gsdmd*^*−/−*^ macrophages favors the idea that GSDMD cleavage occurs upstream of mROS production during *B. cenocepacia* infection.

Inflammasome activation can be affected by *B. cenocepacia* secretory systems (SS) through which, *B. cenocepacia* is able to secrete effector proteins inside the host cytosol^12,56,102^. T6SS, but not T3SS, was identified to be responsible in eliciting pyrin-dependent, inflammasome activation within human monocytes and THP-1 cells^56^. Furthermore, T3SS plays a role in IL-1β maturation^13,103^, in addition to its role in maintaining *B. cenocepacia* survival and replication in murine macrophages^12^. Here, we have demonstrated that, T3SS, T4SS, and T6SS of *B. cenocepacia* contribute to the cleavage of GSDMD, CASP1, and IL-1β in *B. cenocepacia* infected macrophages. Moreover, all of these SSs promote intracellular bacterial persistence within murine macrophages. These findings clarify the different activators and factors that affect inflammasome activation in response to *B. cenocepacia* infection.

Taken together, GSDMD mediates a variety of double-edged functions that may affect bacterial infection and inflammation in a contrasting manner. The recognition that GSDMD exerts cellular functions independently of cell death is gaining momentum and warrants further research.

### Future directions

The interplay between mROS, GSDMD and autophagy during different infections may differ which licenses more investigation.

## Materials and methods

### Bacterial strains

*Burkholderia cenocepacia* (*B. cenocepacia*) K56–2 is a clinical isolate obtained from a CF patient who is not directly involved in the study^104^. Its derivate MH1K is a gentamicin-sensitive strain^105^. *B. cenocepacia* K56–2, used in immunofluorescence experiments, is complemented with a plasmid for red fluorescent protein (Ds-Red). *B. cenocepacia* ΔT3SS, ΔT4SS-1, and ΔT6SS mutants were kindly provided by Dr. Valvano at Queen’s University, Belfast, UK. All bacterial strains were grown overnight in LB medium at 37 °C and 200 rpm as previously described^59,64,65,105^.

### Mice

C57BL/6 wild-type (WT) mice were obtained from the Jackson Laboratory (Bar Harbor, ME, USA). *Casp11*^*−/−*^ mice were generously given by Dr. Yuan at Harvard Medical School, Boston, MA, USA^106^. *Gsdmd*^*−/−*^ mice were a gift from Dr. Thirumala-Devi Kanneganti at St. Jude Children’s Research Hospital, Memphis, TN, USA. *Casp1*^*−/−*/casp11Tg^ mice were kindly provided by Dr. Vishva Dixit at Genentech, San Francisco, CA, USA. All mice were housed in a pathogen-free facility, and experiments were conducted with approval from the Animal Care and Use Committee at the Ohio State University (Columbus, OH, USA) which is accredited by AAALAC International according to guidelines of the Public Health Service as issued in the Guide for the Care and Use of Laboratory Animals (revised 1996).

### Bone marrow-derived macrophages

For the generation of primary bone marrow-derived macrophages (BMDMs) from mice, tibias and femurs were flushed with IMDM medium (Thermo Fisher Scientific, 12440053) supplemented with 10% fetal bovine serum (Thermo Fisher Scientific, 16000044), 50% L cell-conditioned medium, 0.6 × MEM non-essential amino acids (Thermo Fisher Scientific, 11140050) and 0.1% penicillin and streptomycin (Thermo Fisher Scientific, 15140122). Cells were cultivated at least 6 days at 37 °C in a humidified atmosphere containing 5% CO_2_ as previously described^12,17,19,105,107^.

### In vitro infection of primary macrophages

Prior to infection, macrophages were cultivated in IMDM medium supplemented with 10% fetal bovine serum for at least 1 h. In vitro infections were performed at MOI10:1, including centrifugation for 5 min at 30×*g* to synchronize the infection. Cells were infected with *B. cenocepacia* MH1K for 1 h followed by 30 min incubation with 50 µg/mL gentamicin (Thermo Fisher Scientific, 15750060) to avoid extracellular bacterial replication. To determine the macrophage associated colony forming unit (CFU), macrophages were lysed using 0.1% Triton X-100 (Fisher Scientific, BP151) in PBS at the indicated time points, and serial dilutions of the lysates were incubated on LB agar for 48 h. To protect against cell death, cells were incubated with 5 mM glycine (VWR Life Science, 76201-024) 1 h prior to infection and continuously until the end of the experiment. To inhibit cellular reactive oxygen species (ROS), macrophages were incubated with 3 mM *N*-Acetyl-l-cysteine (NAC) (ALX-105-005-G005) which was added 1 h post-infection and continuously until the end of the experiment. On the other hand, to specifically inhibit mitochondrial ROS, cells were incubated with 20 µM Mitotempo (Millipore Sigma, SML0737) which was added 1 h prior to infection and continuously until the end of the experiment. Additionally, to specifically induce mitochondrial ROS, cells were incubated with 1 µM MitoPQ (Abcam, ab146819). Furthermore, to inhibit autophagy in order to determine the autophagy flux, the cells were incubated with 100 nM Bafilomycin A1 (Baf A1) (Enzo, BML-CM110-0100) which was added 2 h before designated time point.

### In vivo infection

For intratracheal infection, mice were anesthetized with Isoflurane and inoculated with 100 µL of phosphate-buffered saline (PBS; Thermo Fisher Scientific, 14190144) containing 10 × 10^6^ CFU/mouse (organ CFU, histology) unless otherwise noted. To determine the bacterial load in organs, mice were sacrificed at 4 h and 48 h post-infection to collect lung, liver, and spleen for homogenization in PBS as previously described^59,107^. Data are presented as CFU per gram of organ. For survival experiments, animals were monitored daily for 6–8 days.

### Phagosome enrichment

Medium was removed from seeded BMDMs (30 × 10^6^ cells) and replaced with ice-cold PBS. Cells were harvested and washed twice with ice-cold PBS, resuspended in homogenization buffer (250 mM sucrose, 0.5 mM EGTA, 20 mM Hepes/KOH (pH 7.2)), and lysed in a Dura Grind stainless-steel homogenizer (VWR, 22877-280). The resulting cell homogenate was cleared by centrifugation, and organelles were fractionated in a discontinuous sucrose density gradient by ultracentrifugation at 100,000×*g*. Phagosomes were recovered from the interface of 55% and 65% sucrose layers, separated through a 15% Ficoll cushion at 18,000×*g*, and concentrated in a final centrifugation step at 18,000×*g*^108,109^.

### Confocal microscopy

Macrophages were cultured on glass coverslips in 24-well plates and fixed with 4% paraformaldehyde for 20 min at the indicated time points. For permeabilization, cells were treated with ice cold methanol for 10 sec followed by 0.1% Triton X-100 for 20 min before blocking with 5% goat serum (Thermo Fisher Scientific, 16,210,064) in PBS. LC3A/B (Cell Signaling Technology, 4108) was visualized using goat anti-rabbit IgG secondary antibody conjugated to Alexa Fluor 488 (Molecular Probes, A-11008). Nuclei were stained with 1 µg/mL of 4ʹ,6ʹ-diamino-2-phenylindole (DAPI; Molecular Probes, D1306,) in PBS-5% goat serum for 15 min. Images were captured using a laser scanning confocal fluorescence microscope with a 60X objective (Olympus Fluoview FV10i) as previously described^19,105^. Intensities of LC3I/II were measured using ImageJ Software as previously described^12^.

### Cytokine and MPO analysis

Cytokines in cell culture supernatants were measured by R&D Systems DuoSet ELISA Development Systems (murine IL-1α, DY400, murine IL-1β, DY401, murine IL-6, DY406, murine CXCL1/KC, DY453, murine TNF-α, DY410 and murine Myeloperoxidase (MPO), DY3667) according to the manufacturer’s instructions.

### Histological analysis

Lungs were removed from infected mice, and fixed in 10% formalin at room temperature. Sample preparation, processing, hematoxylin & eosin staining, and semi-quantitative slide evaluation using ordinal grading scales was performed by the Histology laboratory within the Department of Veterinary Biosciences at The Ohio State University as previously described^59^.

### Immunoblotting

Protein extraction from macrophages was performed using TRIzol reagent (Thermo Fisher Scientific, 15596026) according to the manufacturer’s instructions. Briefly, after phase separation using chloroform, 100% ethanol was added to the interphase/phenolchloroform layer to precipitate genomic DNA. Subsequently, the phenol-ethanol supernatant was mixed with isopropanol to isolate proteins. The Bradford method was used to determine protein concentrations in the cell lysate. Equal amounts of protein were separated by SDS-PAGE and transferred to a polyvinylidene fluoride (PVDF) membrane. Membranes were incubated overnight with antibodies against GSDMD (Abcam, ab209845), CASP11 (abcam, ab180673), CASP1 (AdipoGen, AG-20B-0042-C100), murine IL-1b (R&D Systems, AF-401-NA), LC3A/B (Cell Signaling Technology, 12741) and GAPDH (Cell Signaling Technology, 2118). Corresponding secondary antibodies conjugated with horseradish peroxidase in combination with enhanced chemiluminescence reagent (Amersham, RPN2209,) were used to visualize protein bands. Densitometry analyses were performed by normalizing target protein bands to their respective loading control (GAPDH) using ImageJ software as previously described^17,19^.

### LDH assay

LDH release from macrophages infected with *B. cenocepacia* was measured using the CytoTox-ONE Homogeneous Membrane Integrity Assay (Promega, G7891) according to the manufacturer’s instructions. *B. cenocepacia*-induced LDH release [%] = ((infected sample-low control)/ (high control-low control))*100 (as described before^12^).

### Mitochondrial ROS assay

To determine mitochondrial superoxide production, macrophages were incubated for 30 min with 2 µM MitoSOX dye (Thermo Fisher Scientific, M36008) diluted in cell imaging solution (formulated as previously described^17^) at 37 °C in a humidified atmosphere containing 5% CO_2_. Then cells were washed with PBS and further incubated with cell imaging solution. The fluorescence was read using a SpectraMax i3x microplate reader at 510/580 nm. Fluorescence values are reported as relative to the no treatment.

### Mitochondrial membrane potential (MMP) assay

To determine mitochondrial membrane potential, macrophages were incubated for 30 min with 10 nM TMRM dye (Molecular Probes, Invitrogen, UK, T668) diluted in cell imaging solution (formulated as previously described^17^) at 37 °C in a humidified atmosphere containing 5% CO2. Then cells were washed with PBS and further incubated with cell imaging solution. As a positive control, cells were treated with 10 µM of FCCP (MMP uncoupler) (Millipore Sigma, C2759). The fluorescence was read using a SpectraMax i3x microplate reader at 548/574 nm. Fluorescence values were normalized to cell number as measured per well using a SpectraMax MiniMax 300 Imaging Cytometer, then reported as fold change to the no treatment.

### *B. cenocepacia* susceptibility to H_2_O_2_

WT or *gsdmd*^*−/−*^ macrophages were infected with *B. cenocepacia* (MOI10) for 6 h in the presence of glycine (5 mM). The infected cells were washed with PBS twice and lysed in PBS plus 2% saponin plus 15% BSA to harvest intracellular bacteria as previously described^30^. Lysates were treated for 30 min with PBS, H_2_O_2_ (2, 0.75, 0.5 mM) before plating them on LB agar plates^30^.

### Statistical analysis

Data were analyzed using GraphPad Prism 8.3.0. All figures display mean and standard error of the mean (SEM) from at least three independent experiments as indicated in the figures’ legends. Comparisons between groups were conducted with either Student’s t-test, one-way or two-way ANOVA (depending on the data structure) followed by Holm’s adjustment for multiple comparisons as indicated^12^. Adjusted P < 0.05 was considered statistically significant.

## Supplementary Information

Supplementary Information.
