# Author Correction: Gasdermin D restricts *Burkholderia cenocepacia* infection in vitro and in vivo

**DOI:** 10.1038/s41598-021-92047-9

**Published:** 2021-06-08

**Authors:** Shady Estfanous, Kathrin Krause, Midhun N. K. Anne, Mostafa Eltobgy, Kyle Caution, Arwa Abu Khweek, Kaitlin Hamilton, Asmaa Badr, Kylene Daily, Cierra Carafice, Daniel Baetzhold, Xiaoli Zhang, Tianliang Li, Haitao Wen, Mikhail A. Gavrilin, Hesham Haffez, Sameh Soror, Amal O. Amer

**Affiliations:** 1 Department of Microbial Infection and Immunity, Infectious Diseases Institute, Ohio State University, Columbus, OH USA; 2 Biochemistry and Molecular Biology Department, Faculty of Pharmacy, Helwan University, Cairo, Egypt; 3 Max Planck Unit for the Science of Pathogens, Berlin, Germany; 4 Department of Biology and Biochemistry, Birzeit University, Birzeit, West Bank Palestine; 5 Center for Biostatistics, Ohio State University, Columbus, OH USA; 6 Department of Internal Medicine, Ohio State University, Columbus, OH USA; 7 Center for Scientifc Excellence “Helwan Structural Biology Research” (HSBR), Helwan University, Cairo, Egypt

Correction to: *Scientific Reports* 10.1038/s41598-020-79201-5, published online 13 January 2021

The original version of this Article contained an error in Figure 6B where were the y-axis numbering “0, 10, 20, 30” was incorrectly given as “0, 1, 2, 3”.

The original Figure 6 and accompanying legend appear below.

**Figure 6:**
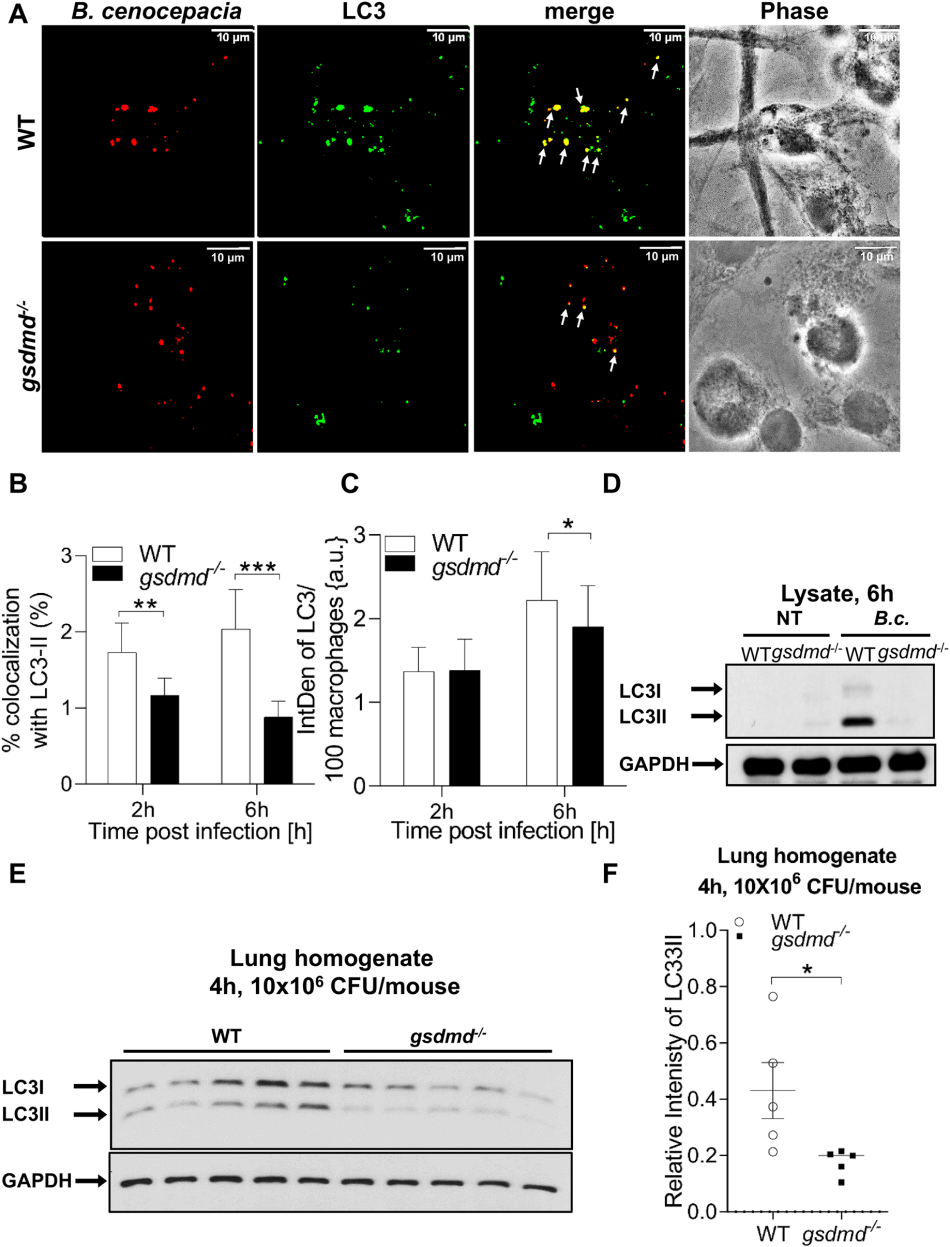
Gsdmd^*−/−*^ mice and their macrophages exhibit less autophagosme formation during *B. cenocepacia* infection. **(A)** Confocal images of LC3 (green) immunofluorescence assay of *B. cenocepacia* (*B.c.*) (red) (MOI10) infected WT and *gsdmd*^*−/−*^ macrophages at 6 h post-infection. White arrows points to *B. cenocepacia*- LC3 co-localization. Scale bar: 10 µM. **(B)** Quantification of *B. cenocepacia* co-localized with LC3 in *B. cenocepacia* infected WT *and gsdmd*^*−/−*^ macrophages treated as in (**A**). Data represent mean ± SEM (n = 5). Statistical analysis was performed using two-way ANOVA. **(C)** Quantification of total LC3 puncta in *B. cenocepacia* infected WT *and gsdmd*^*−/−*^ macrophages treated as in (**A**) using ImageJ Software. Values are mean percentage ± SEM calculated by scoring 96 randomly chosen fields of view from 6 independent experiments. Statistical analysis was performed using paired two tailed Student’s t-test. **(D)** Immunoblot analysis of LC3 in whole cell lysate separated of WT and *gsdmd*^*−/−*^ macrophages treated as in (**A**). **(E)** Immunoblot analysis of LC3 in lung homogenate separated of WT and *gsdmd*^*−/−*^ mice after being infected intratracheally for 4 h by *B. cenocepacia* (10 × 10^6^ CFU/mouse) (n = 5). **(F)** Densitometry analysis of immunoblots represented in (**E**). Data represent mean ± SEM (n = 5). Statistical analysis was performed using unpaired two-tailed student’s t-test *p ≤ 0.05, **p ≤ 0.01, ***p ≤ 0.001.

The original Article has been corrected.

