# Supplementary material for: Gasdermin D restricts Burkholderia cenocepacia infection in vitro and in vivo
Source: Sci Rep. 2021 Jan 13;11:855. doi: 10.1038/s41598-020-79201-5 (PMC7807041; doi:10.1038/s41598-020-79201-5)

# Gasdermin D restricts *Burkholderia cenocepacia* infection *in vitro* and *in vivo*


## Authors:

Shady Estfanous[1,2], Kathrin Krause[1,3], Midhun N K Anne[1], Mostafa Eltobgy[1], Kyle Caution[1], Arwa Abu Khweek[1,4], Kaitlin Hamilton[1], Asmaa Badr[1], Kylene Daily[1], Cierra Carafice[1], Daniel Baetzhold[1], Xiaoli Zhang[5], Tianliang Li[1], Haitao Wen[1], Mikhail A. Gavrilin[6], Hesham Haffez[2,7], Sameh Soror[2,7] and Amal O. Amer[1]

## Affiliations:

[1]Department of Microbial Infection and Immunity, Infectious Diseases Institute, Ohio State University, Columbus, OH, USA.

[2]Department of Biochemistry and Molecular Biology, Faculty of Pharmacy, Helwan University, Egypt.

[3]Max Planck Unit for the Science of Pathogens, Berlin, Germany.

[4]Department of Biology and Biochemistry, Birzeit University, Birzeit, West Bank, Palestine.

[5]Center for Biostatistics, Ohio State University, Columbus, OH, USA.

[6]Department of Internal Medicine, Ohio State University, Columbus, OH, USA.

[7]Center of Excellence, Helwan Structure Biology Research, Cairo, Egypt.


# Supplementary Figures

Supplemental Figure1 – Related to Figure 1

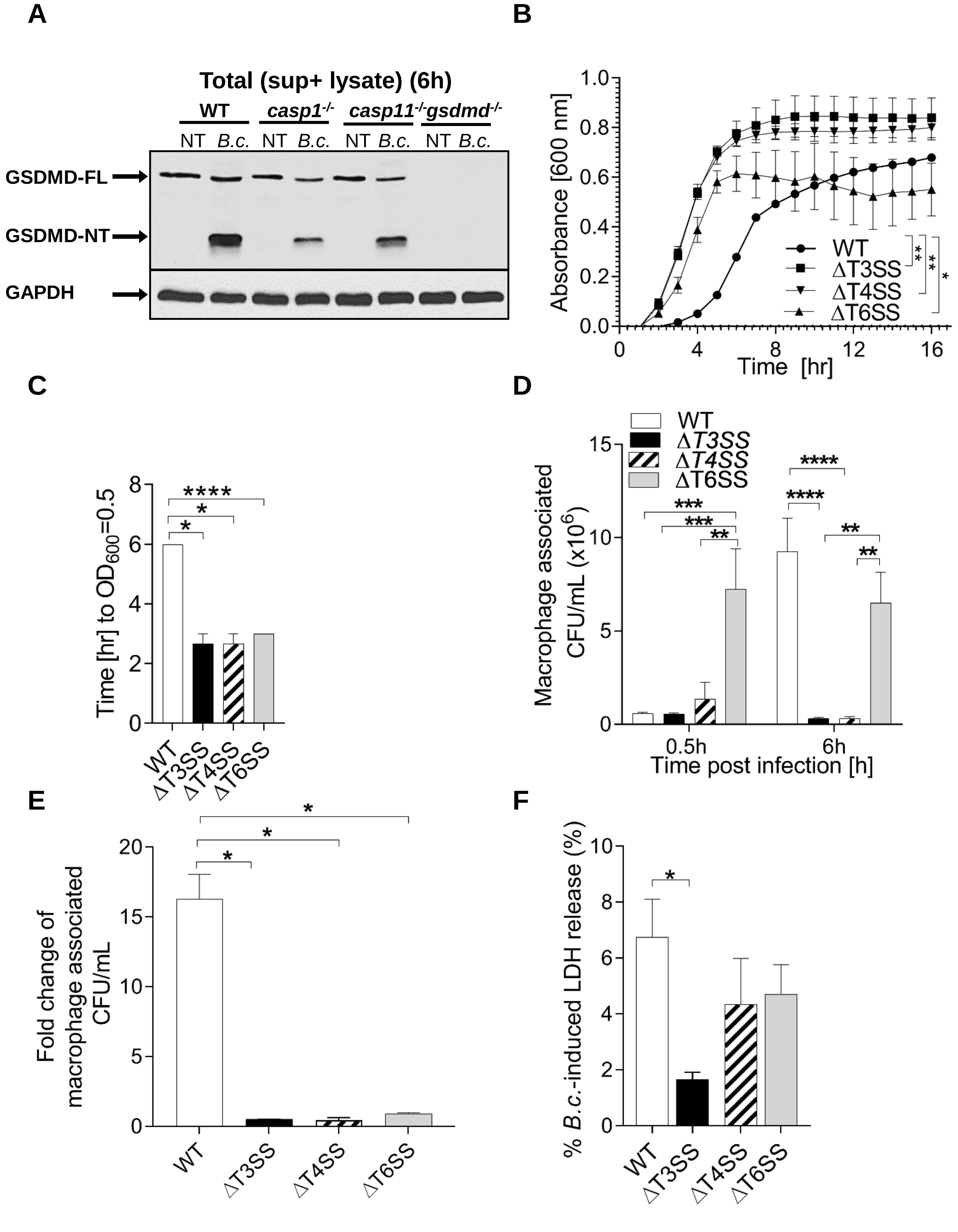

**Supplemental Figure1 – (Related to Figure 1): CASP11 and, CASP1 influence GSDMD cleavage in *B. cenocepacia* infected murine macrophages. Bacterial secretion systems are required for bacterial growth in macrophages.**

**(A)** Immunoblot analysis of GSDMD and GAPDH in total cell lysate and cell supernatant from WT, *casp1*$^{-/-}$, *casp11*$^{-/-}$, and *gsdmd*$^{-/-}$ macrophages infected with *B. cenocepacia* (*B.c.*) (MOI10) at 6h post-infection. **(B)** Growth curve of WT *B. cenocepacia*, ΔT3SS*,* ΔT4SS, and ΔT6SS mutants incubated in LB media (n=3). Statistical analysis was performed using two-way ANOVA. **(C)** Time required to reach absorbance of $OD_{600}$=0.5 for the growth curve done in (B). **(D)** Macrophage associated CFUs of WT *B. cenocepacia,* ΔT3SS*,* ΔT4SS, *and* ΔT6SS mutants (MOI10) in WT macrophages. Data represent mean ± SEM (n=5). Statistical analysis was performed using two-way ANOVA. **(E)** Fold change increase of the CFU performed in (D) at 6h relative to CFU performed at 0.5h. **(F)** WT *B. cenocepacia,* ΔT3SS*,* ΔT4SS *and* ΔT6SS mutants-induced cytotoxicity were calculated by measuring LDH release in supernatants at 6h post-infection of WT macrophages (MOI10) (n=3). Statistical analysis was performed using one-way ANOVA. *p≤0.05, **p≤0.01, ***p≤0.001.

Supplemental Figure 2 – Related to Figure 3

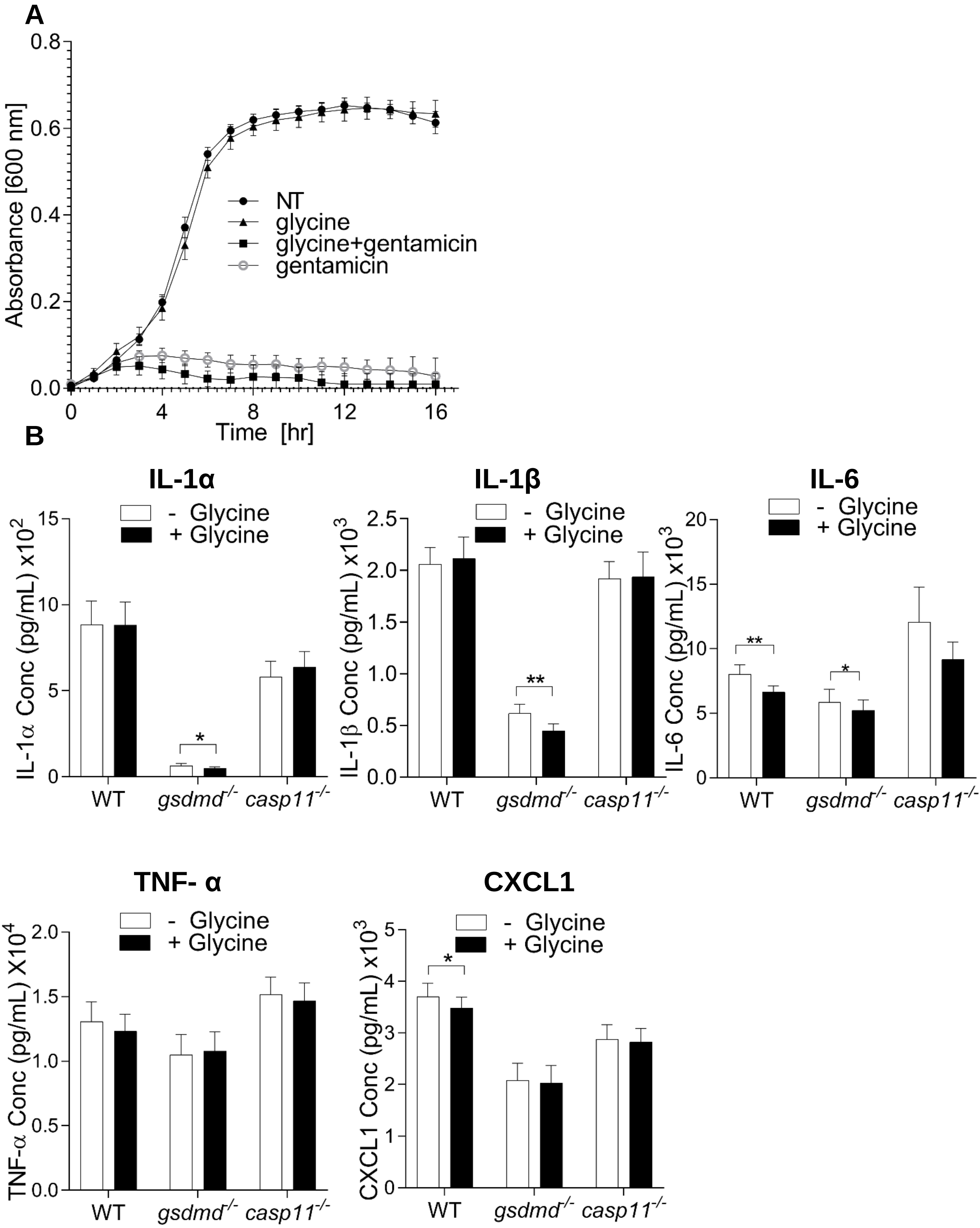

**Supplemental Figure 2 – (Related to Figure 3): GSDMD promotes the cytokines secretion during *B. cenocepacia* infection independently of cell death.**

**(A)** Growth curve of WT *B. cenocepacia* in LB media in the presence of glycine (5mM), gentamycin and glycine together with gentamycin (n=6). Statistical analysis was performed using two-way ANOVA. NT= no treatment. **(B)** Cytokines release from WT, *gsdmd$^{-/-}$and casp11$^{-/-}$* macrophages infected with *B. cenocepacia* (MOI10) at 6h post-infection in the absence or presence of glycine (5mM). Data represent mean ±SEM (n=20). Statistical analysis was performed using paired two-tailed student's t-test. *p≤0.05, **p≤0.01, ***p≤0.001.

Supplemental Figure 3 – Related to Figure 4

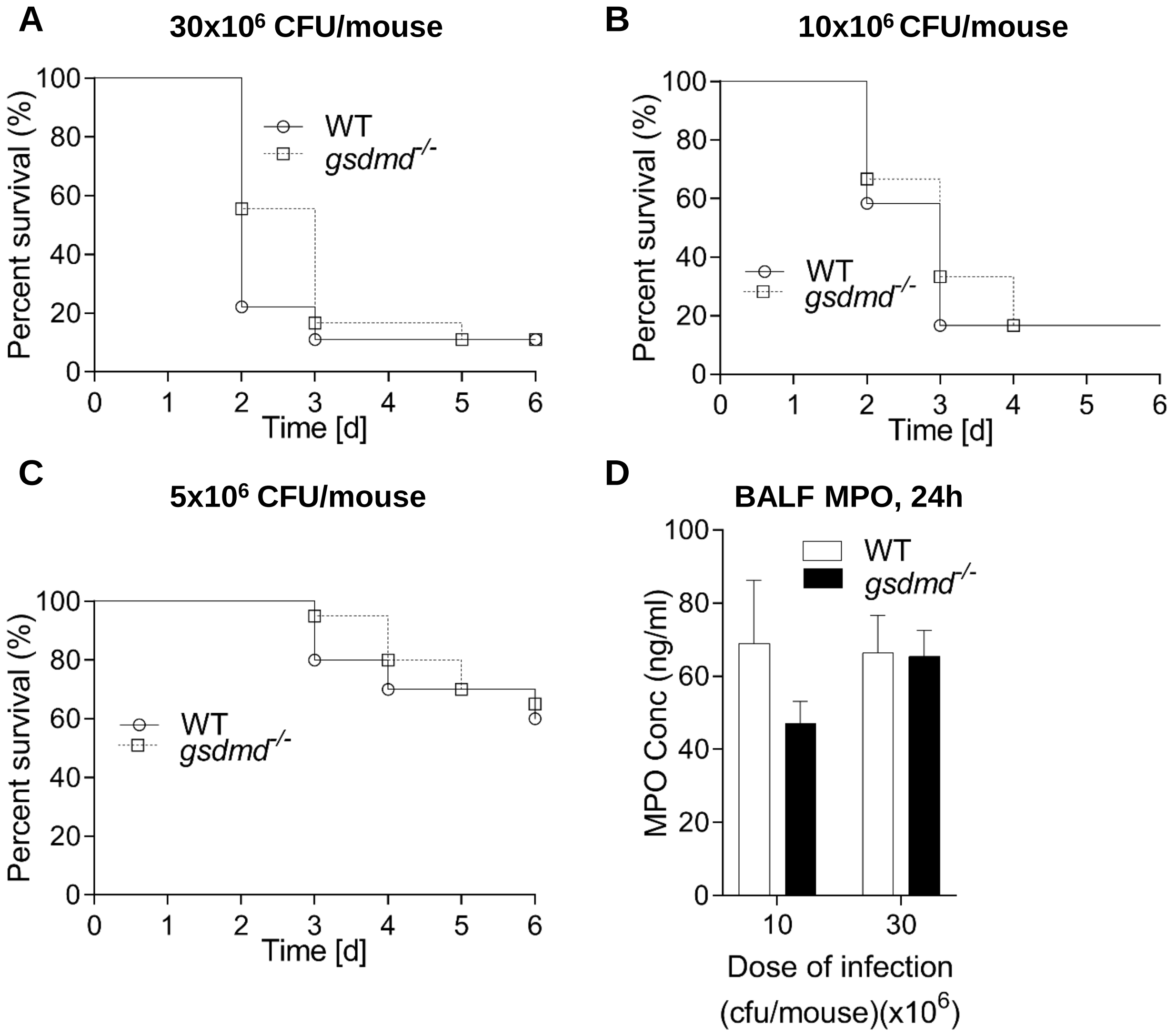

**Supplemental Figure 3 – (Related to Figure 4): GSDMD does not contribute to the mice survival during *B. cenocepacia* infection.**

**(A)** Survival of WT and *gsdmd*$^{-/-}$ mice after intratracheal infection with $30\times10^6$ CFU/mouse. Pooled data from 2 independent experiments (n=18). **(B)** Survival of WT, *gsdmd*$^{-/-}$ , and *casp11*$^{-/-}$ mice after intratracheal infection with $10\times10^6$ CFU/mouse. Pooled data from 2 independent experiments (n=12). **(C)** Survival of WT and *gsdmd*$^{-/-}$ mice after intratracheal infection with $5\times10^6$ CFU/mouse. Pooled data from 2 independent experiments (n=20). Statistical analyses for the survival studies (Fig. SA-C) were performed using Log-rank (Mantel-Cox) test. **(D)** Myeloperoxidase **(**MPO) level at 24h in the bronchoalveolar lavage fluid (BALF) of WT and *gsdmd*$^{-/-}$ mice after intratracheal infection with both $10\times10^6$, and $30\text{x}10^6$ CFU/mouse. Statistical analysis was performed using two-way ANOVA. *$p\leq0.05$, **$p\leq0.01$, ***$p\leq0.001$.

Supplemental Figure 4 – Related to Figure 5

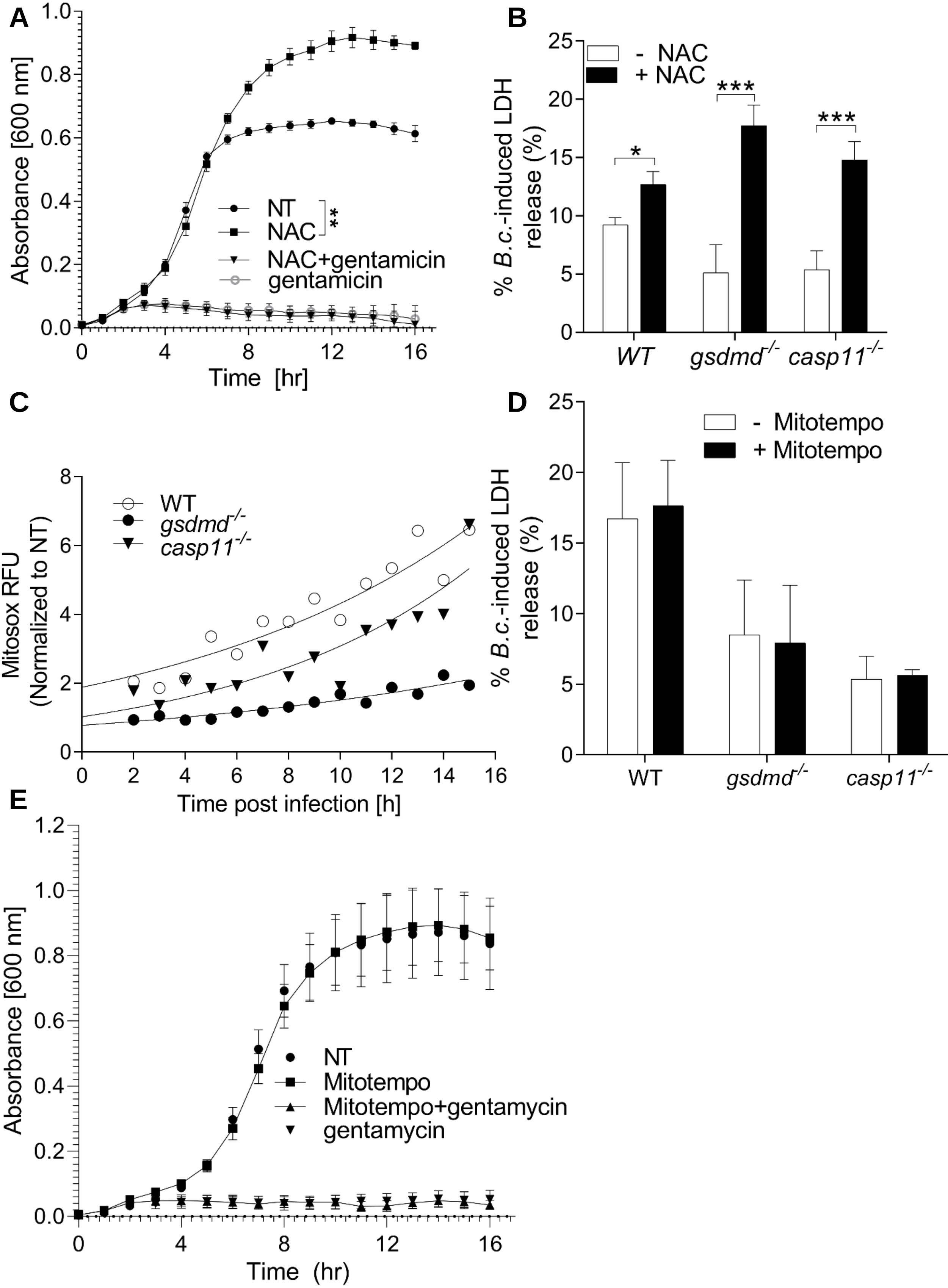

**Supplemental Figure 4 – (Related to Figure 5): N-acetyl cysteine but not Mitotempo increases LB growth of *B. cenocepacia* and cell death.**

**(A)** Growth curve of WT *B. cenocepacia* in LB media in the presence of N-acetylcysteine (NAC), gentamycin, and NAC together with gentamycin (n=6). Statistical analysis was performed using two-way ANOVA. Statistical analyses were performed using two-way ANOVA. NT=no treatment. **(B)** *B. cenocepacia* -induced cytotoxicity was calculated by measuring LDH release in supernatants at 6h post-infection from WT, *gsdmd$^{-/-}$*, and *casp11$^{-/-}$* macrophages, in presence or absence of NAC (3mM) (n=3). Statistical analysis was performed using two-way ANOVA. **(C)** Representative fit curve of the Mitosox assay performed in WT, *gsdmd$^{-/-}$, and casp11$^{-/-}$* macrophages infected with *B. cenocepacia* (MOI10) for 6h post-infection in presence of glycine. **(D)** *B. cenocepacia* -induced cytotoxicity was calculated by measuring LDH release in supernatants from WT, *gsdmd$^{-/-}$,* and *casp11$^{-/-}$* infected as in (B), in presence and absence of Mitotempo (20μM) (n=3). Statistical analyses were performed using two-way ANOVA. **(E)** Growth curve of WT *B. cenocepacia* in LB media in the presence of Mitotempo, gentamycin, and Mitotempo together with gentamycin (n=7). Statistical analysis was performed using two-way ANOVA. NT=no treatment. *p≤0.05, **p≤0.01, ***p≤0.001.

Supplemental Figure 5 – Related to Figure 6

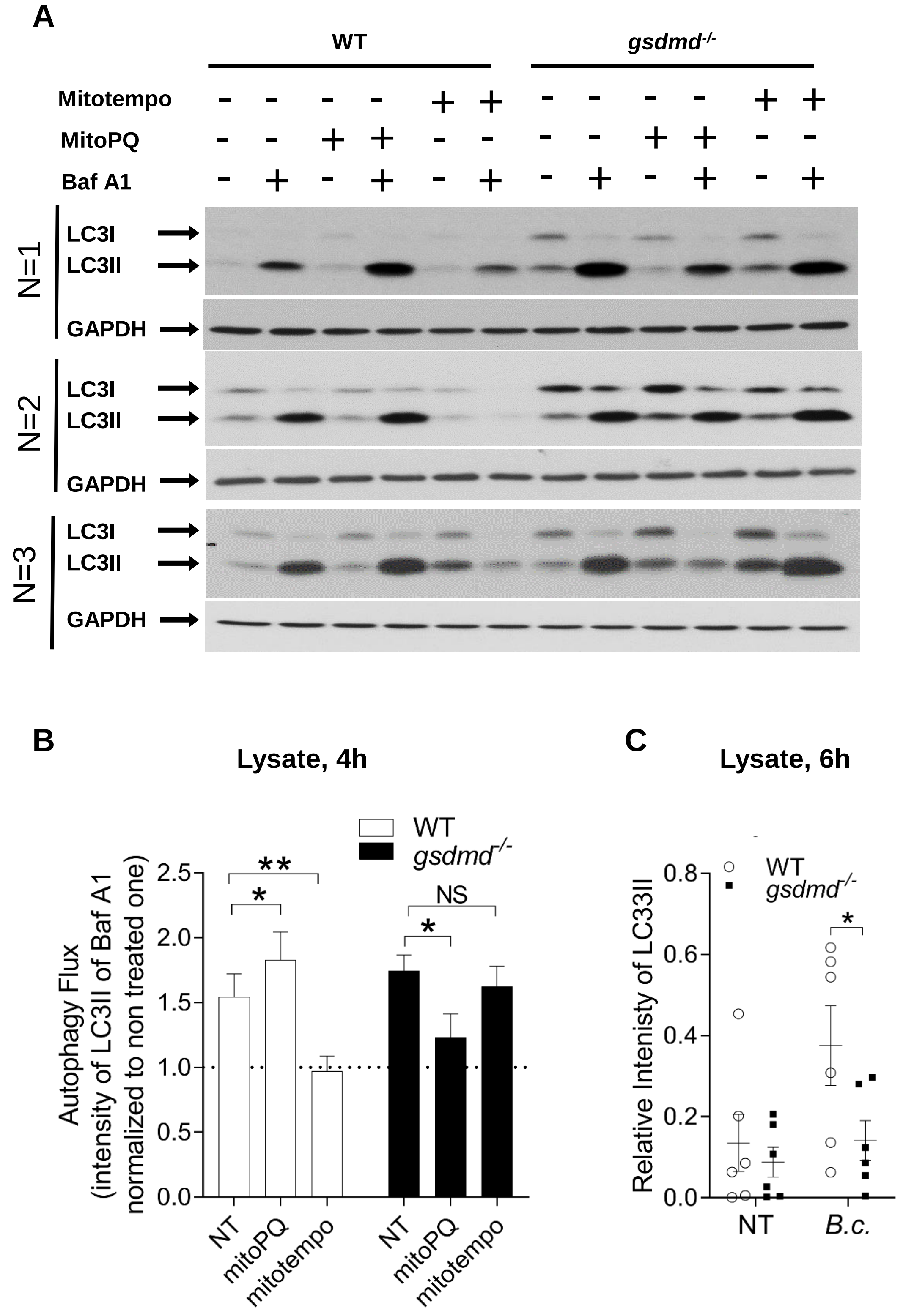

**Supplemental Figure 5 – (Related to Figure 6): mROS and GSDMD positively correlate with autophagosomes formation *in vitro*.**

**(A)** Immunoblot analysis of LC3 and GAPDH in cell lysate to determine autophagy flux in WT, *gsdmd*$^{-/-}$ macrophages at 4h post-treatment with selective mROS inducer (MitoPQ, 1µM) and inhibitor (Mitotempo, 20µM) in presence and absence of autophagy flux inhibitor (Bafilomycin A1) (BafA1) (100nM) which was added 2 hours before the designated time point (n=3)**. (B)** Densitometry analysis of the immunoblots shown in (A). Autophagy flux was measured by normalization of the LC3II intensity in whole cell lysates of BafA1 treated to non-BafA1 treated cells after been normalized to GAPDH. Data represent mean ±SEM (n=3). Statistical analysis was performed using two-way ANOVA. **(C)** Densitometry analysis of immunoblots represented in (Fig. 6D). Data represent mean ±SEM (n=6). Statistical analysis was performed using two-way ANOVA. *B.c.*= *B. cenocepacia*. *p≤0.05, **p≤0.01, ***p≤0.001.

# Full-length gels and blots of the cropped blots in the main figures

## Figure 1A GSDMD N=4

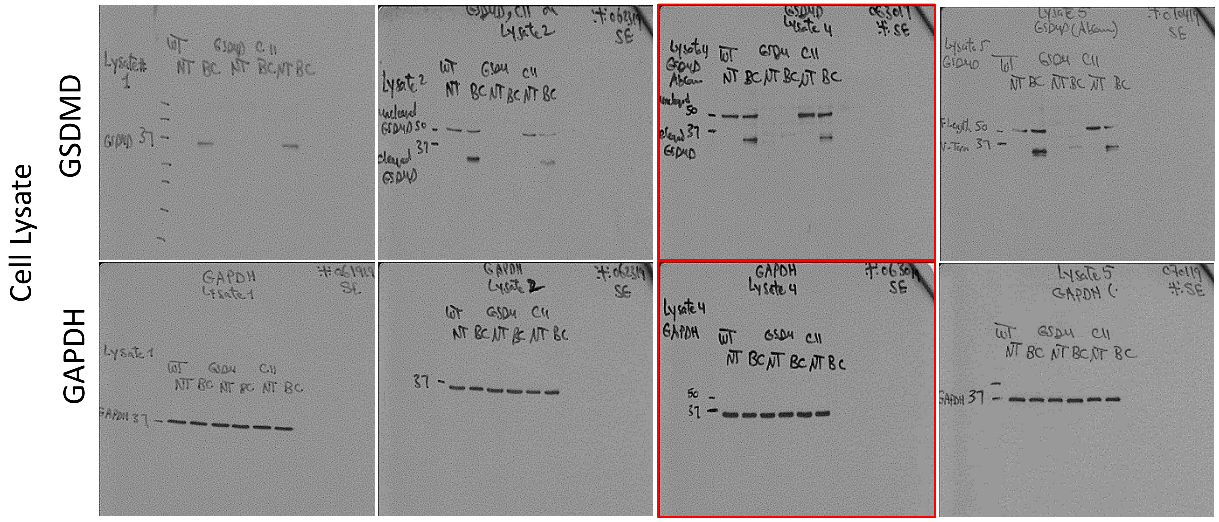


## Figure 1A CASP8 N=3

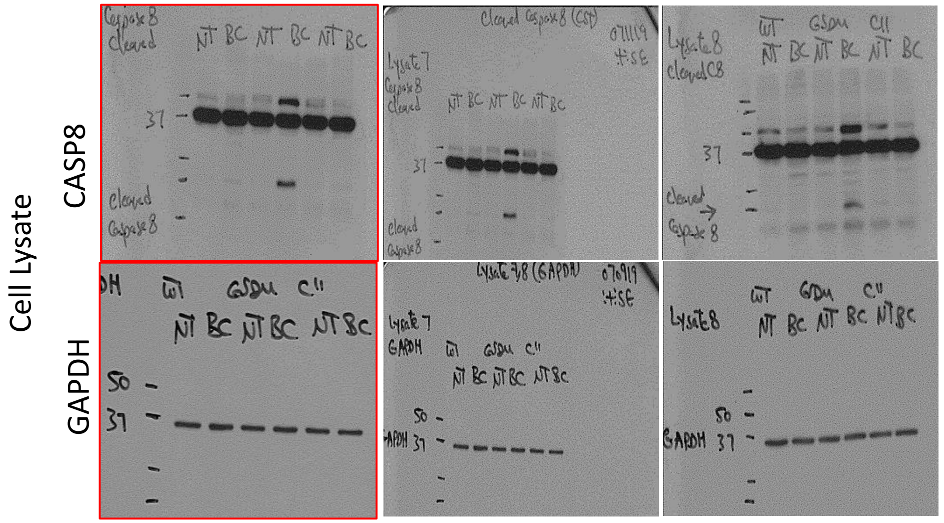


## Figure 1A Uncleasved Caspase 11 N=3

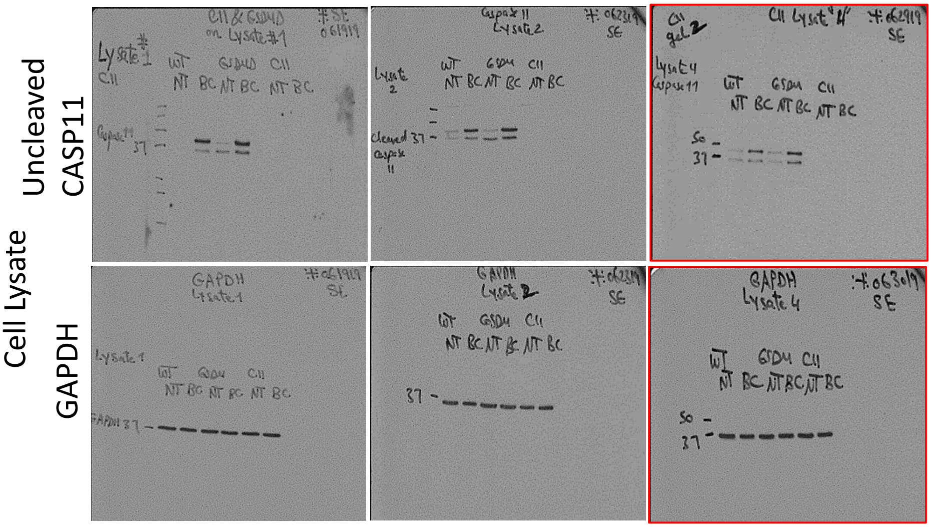

## Figure 1A Cleasved Caspase 11 N=4

Cell Lysate

Cleaved CASP11

GAPDH

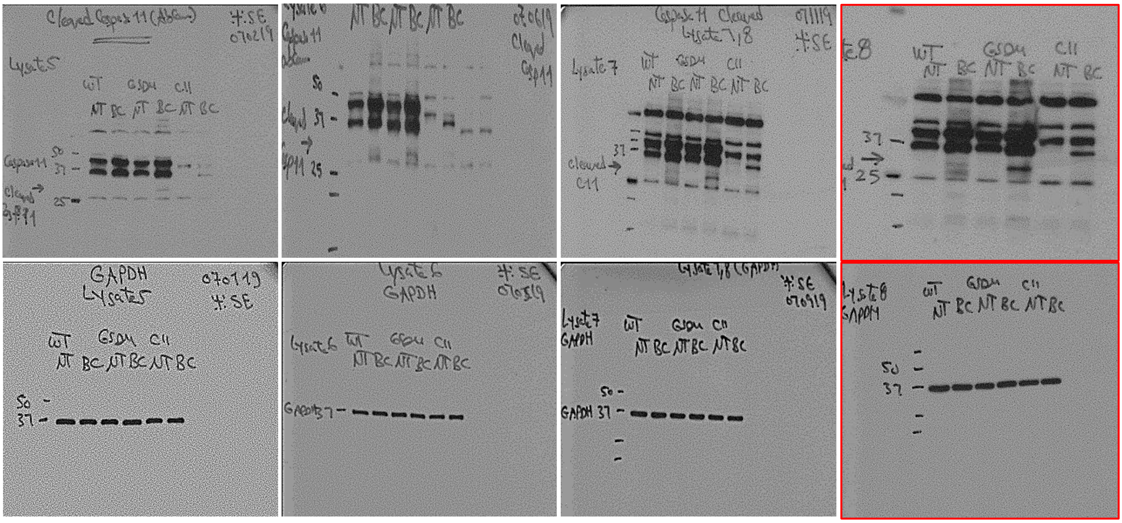

## Figure 1A IL-1β N=4

Cell Lysate

IL-1β

GAPDH

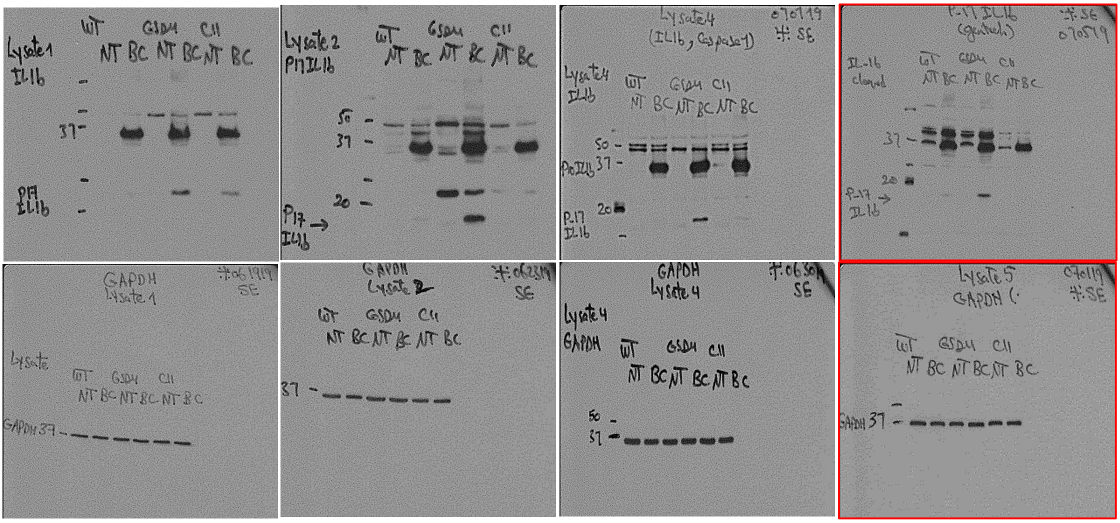

## Figure 1A Cleaved CASP1 N=4

Cell Lysate

CASP1

GAPDH

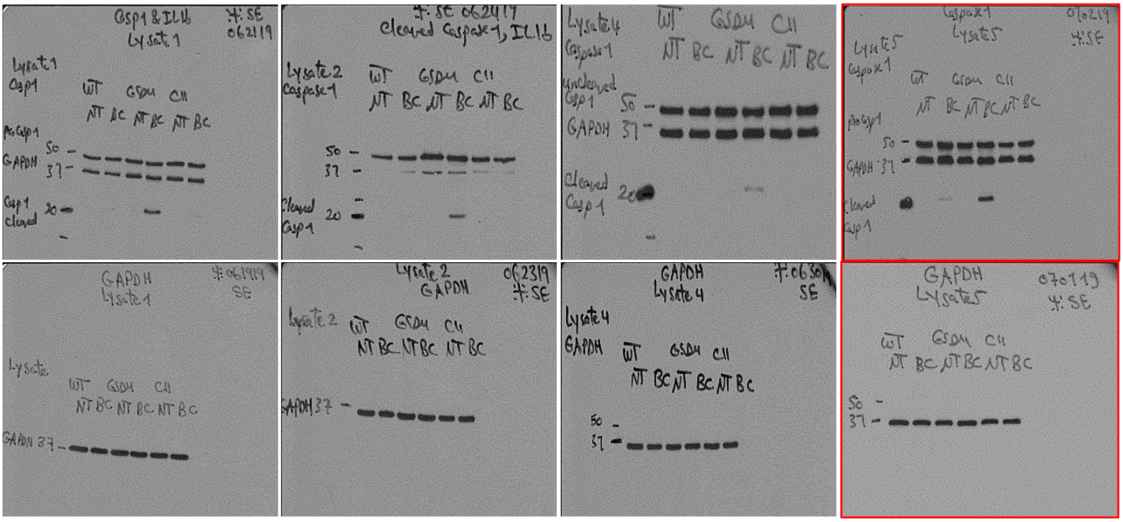

# Figure 1B GSDMD (Sup) N=3

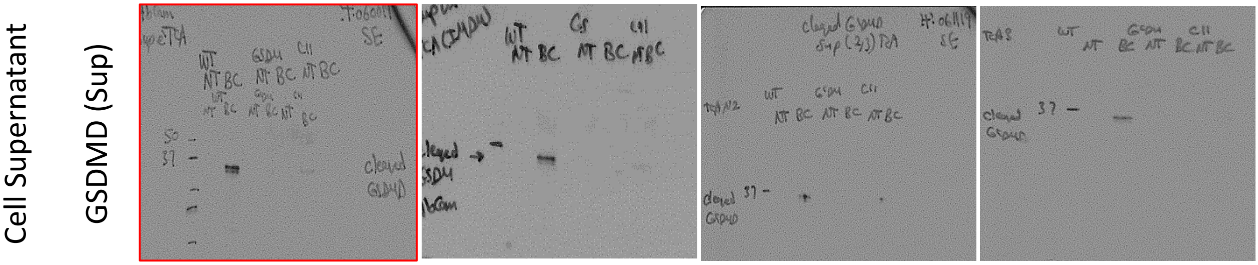


# Figure 1B IL-1β(Sup) N=5

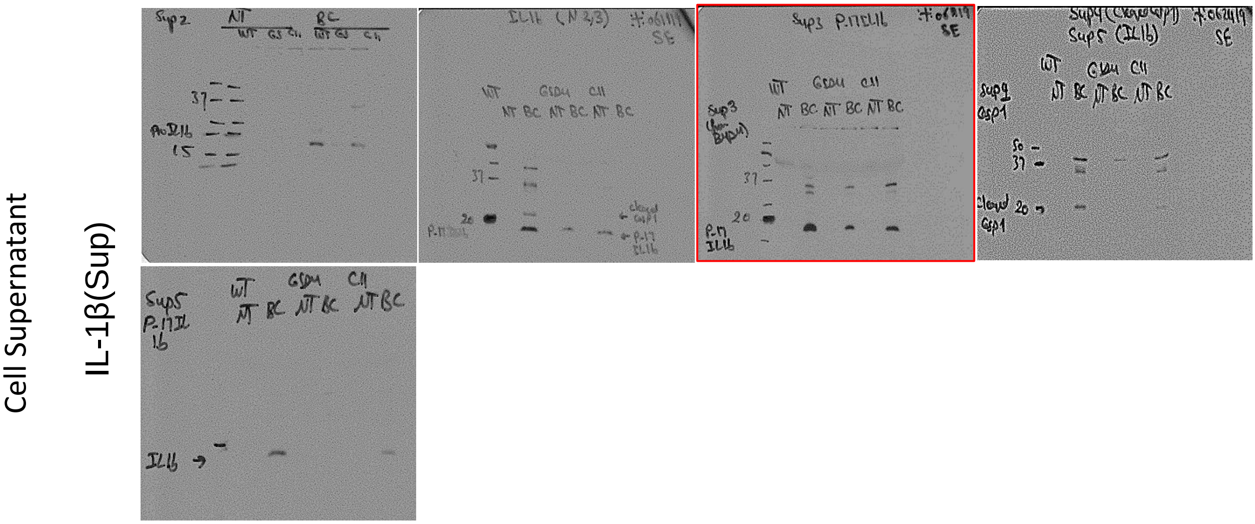


# Figure 1B CASP1(Sup) N=7

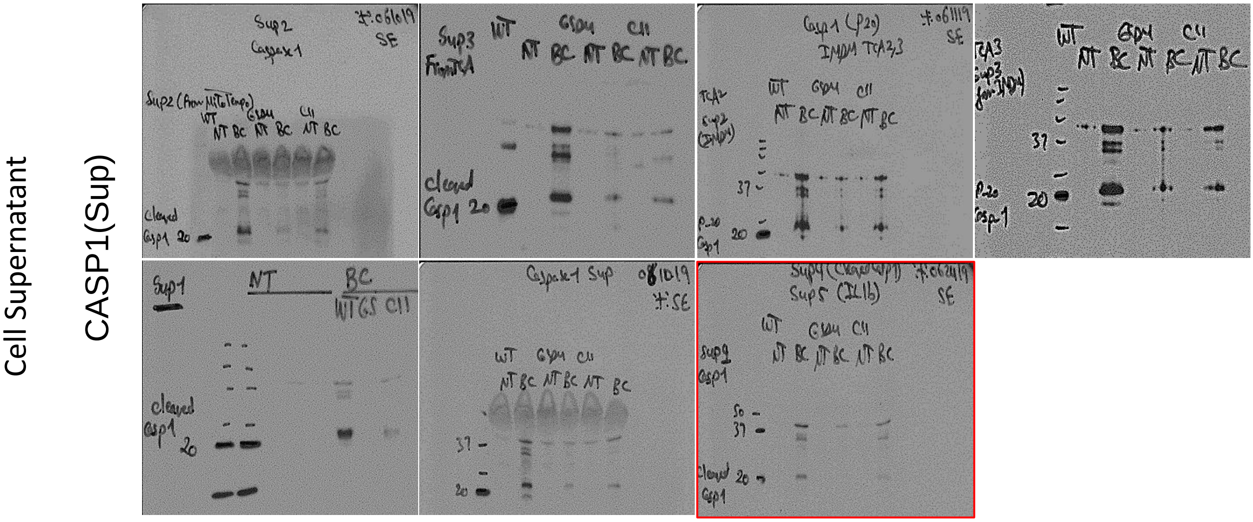

Figure 1B CASP11(Sup) N=6

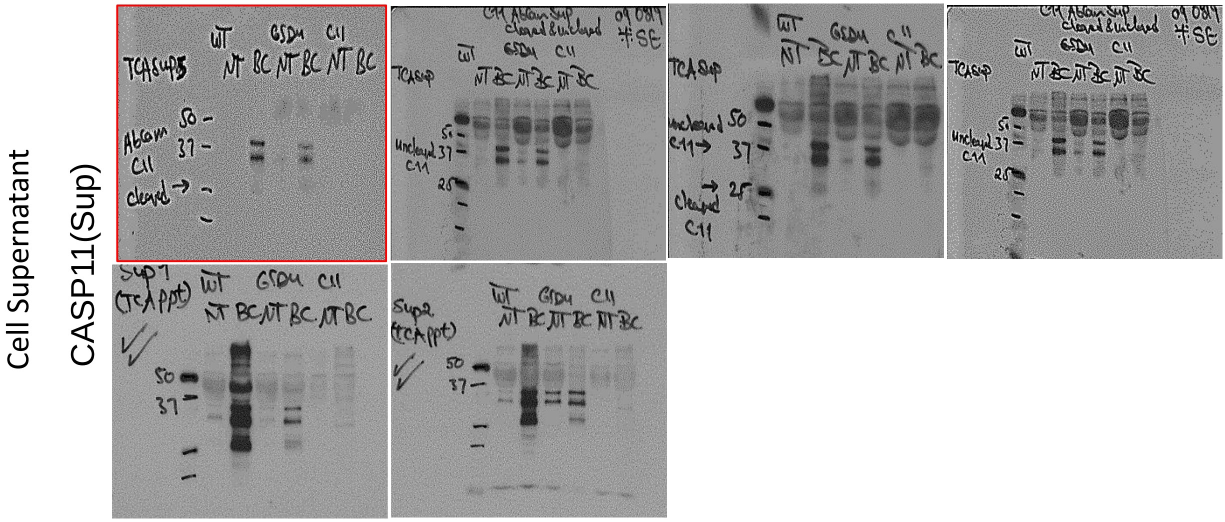


Figure 1C GSDMD N=7

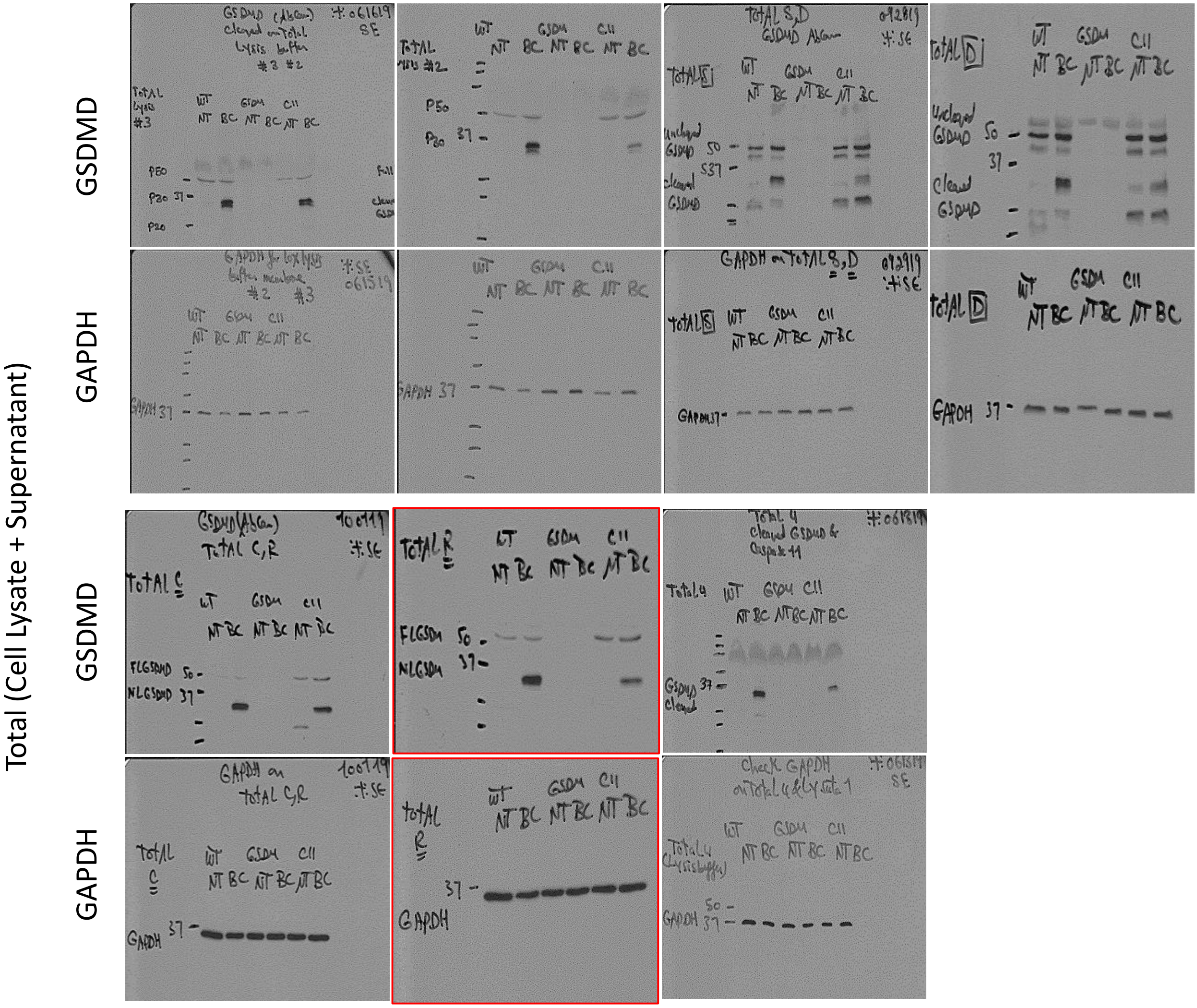

# Figure 1C Uncleaved CASP11 N=7

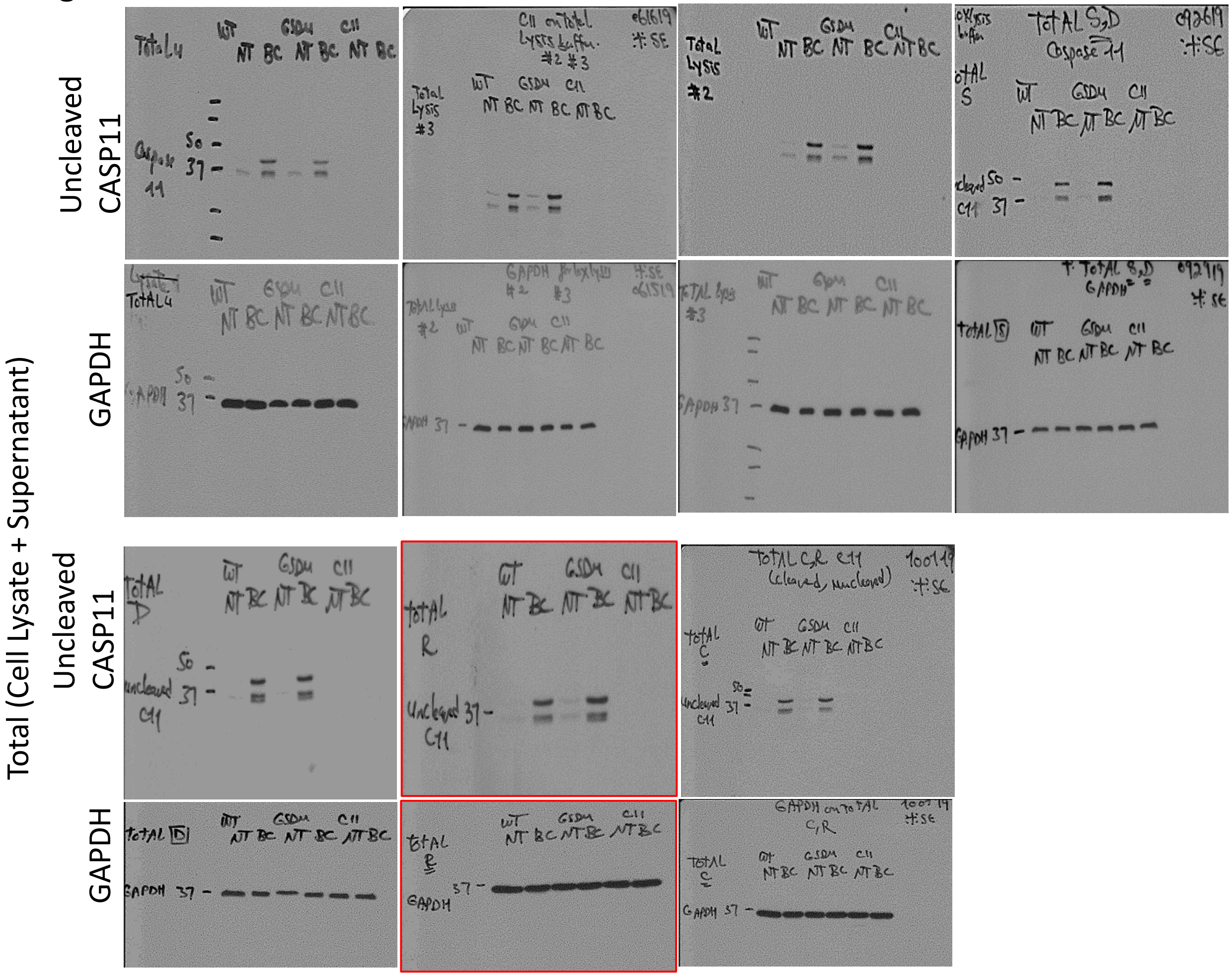


# Figure 1C Cleaved CASP11 N=6

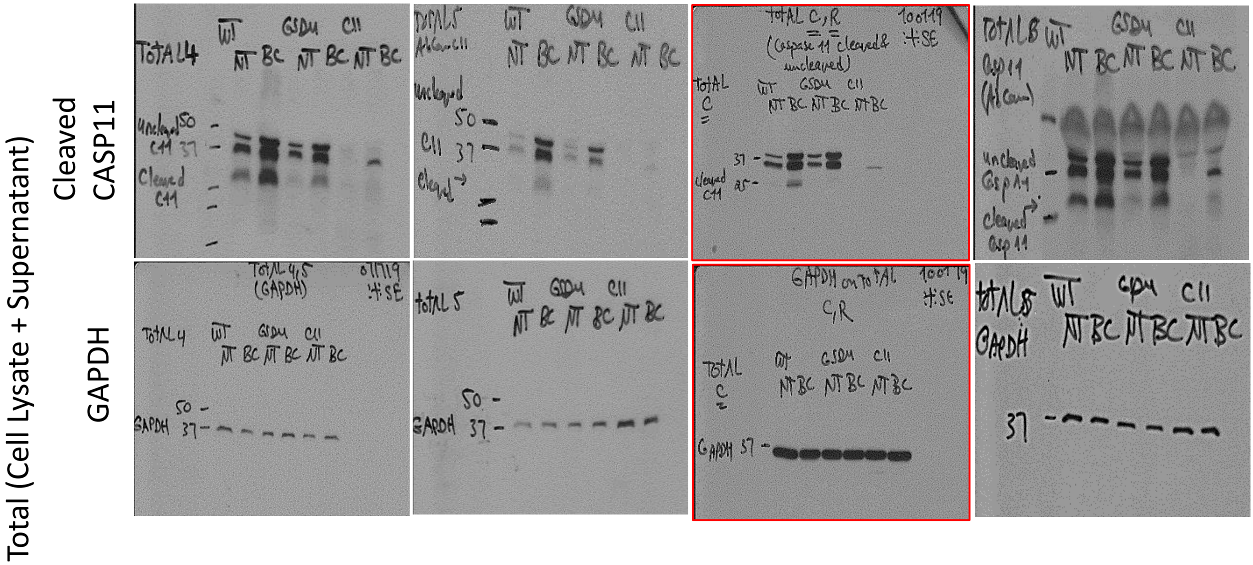

## Figure 1C Cleaved CASP11 N=6 (continue)

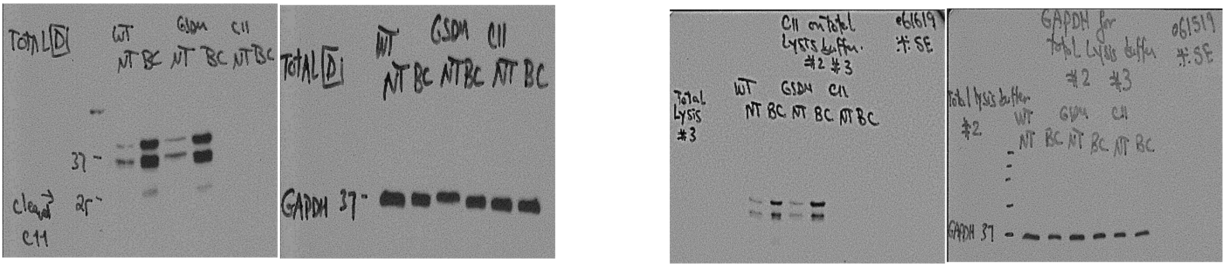

## Figure 1C CASP1 N=7

Total (Cell Lysate + Supernatant)

CASP1

GAPDH

CASP1

GAPDH

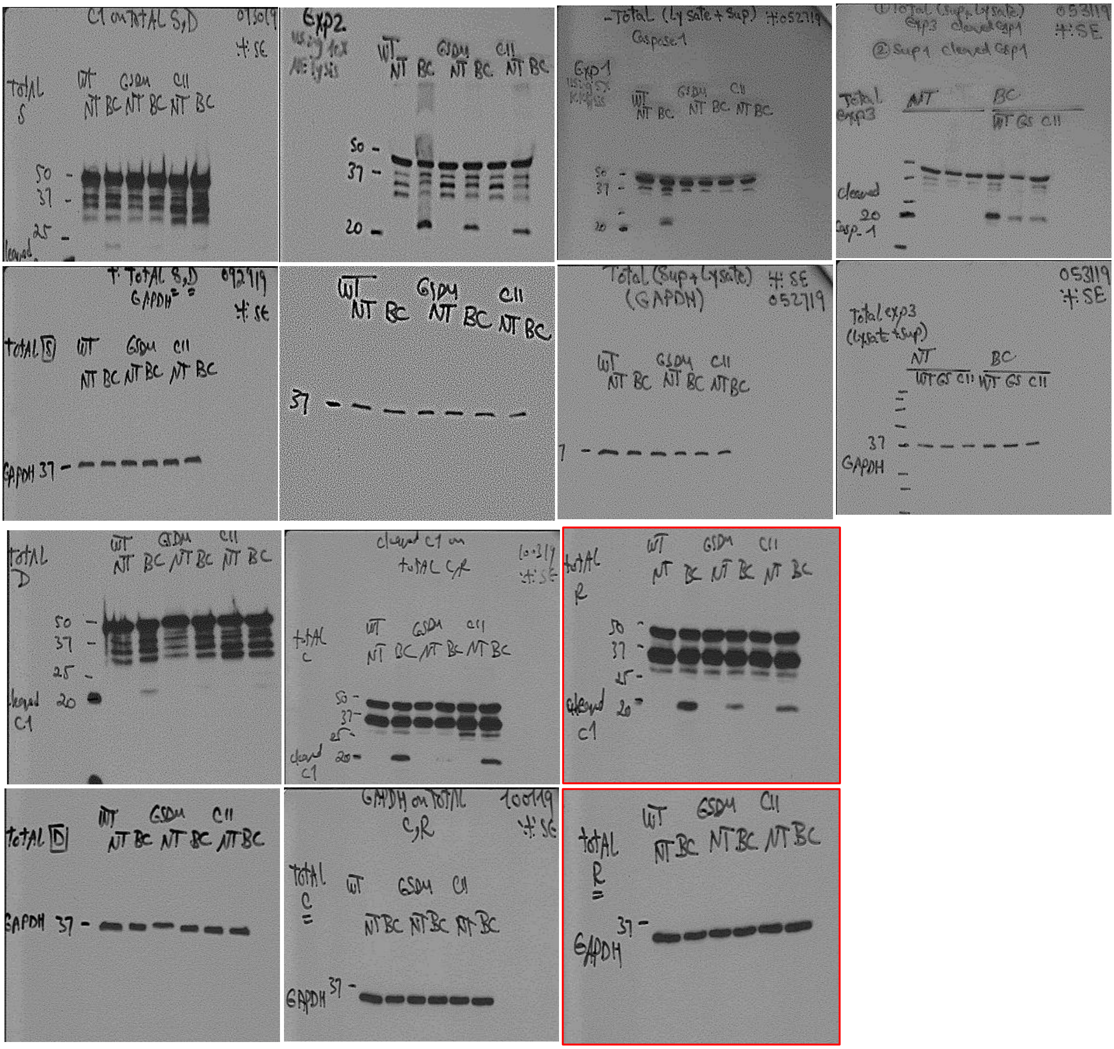

# Figure 1C IL-1β N=7

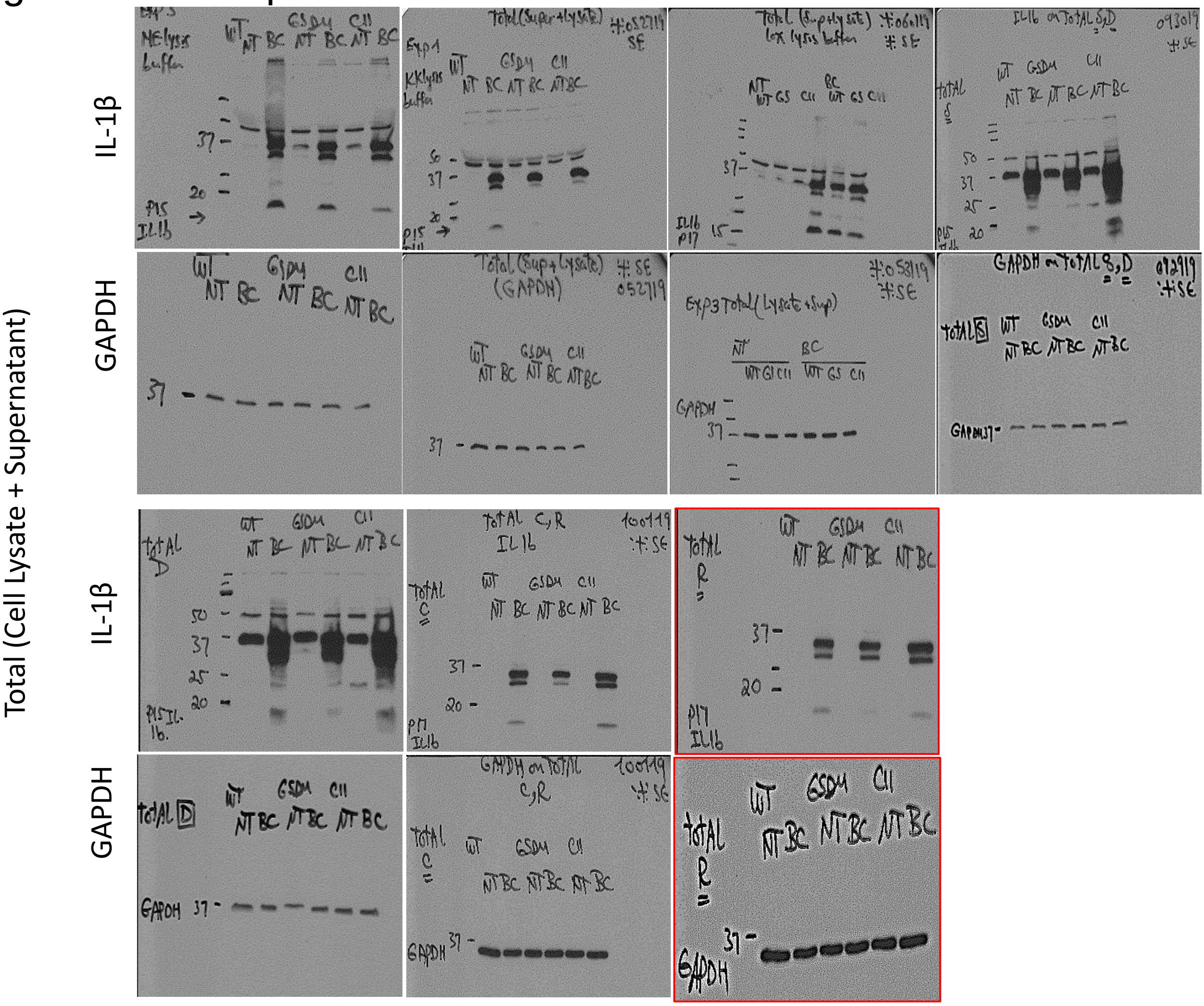


# Figure 1C CASP-8 N=3

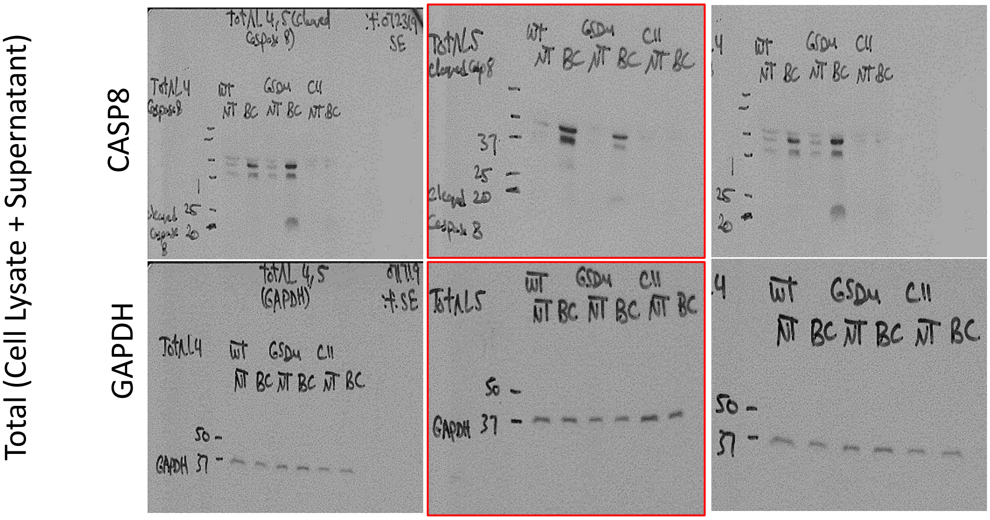

## Figure 1D GSDMD N=4

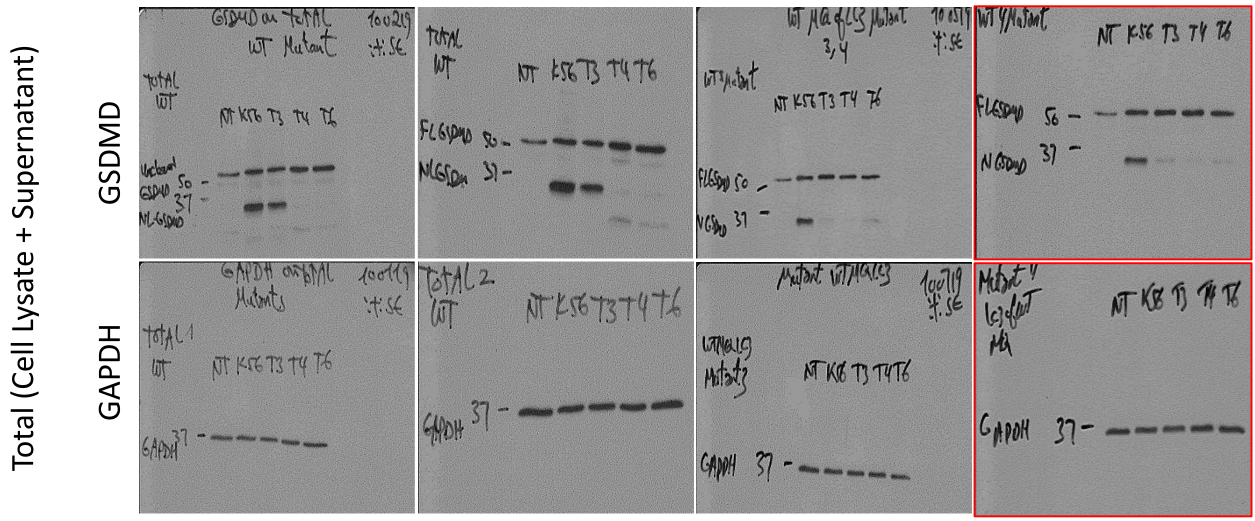


## Figure 1D Uncleaved CASP11 N=4

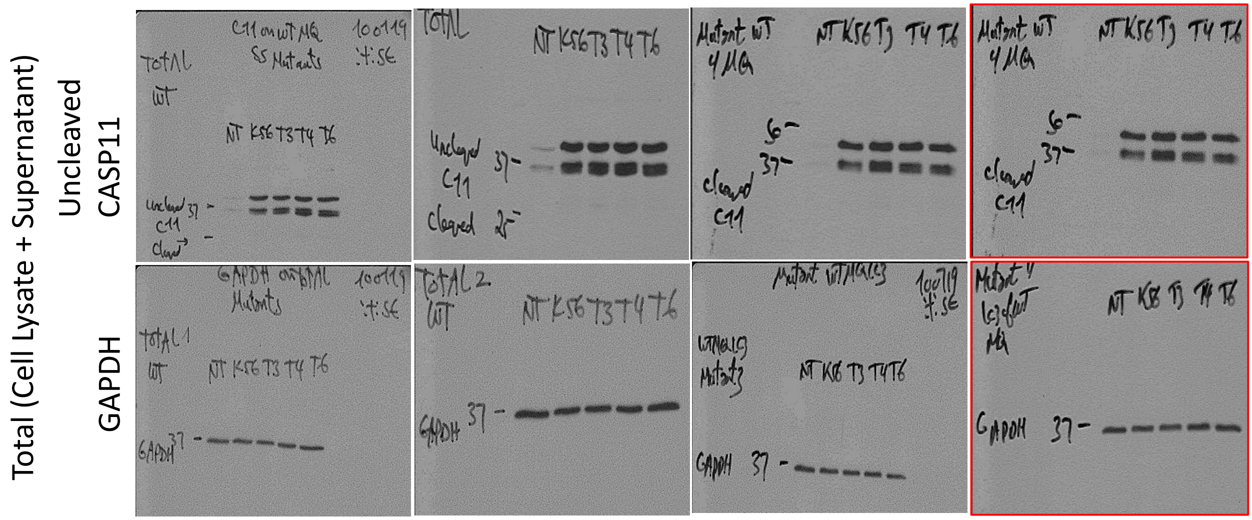


## Figure 1D Cleaved CASP11 N=4

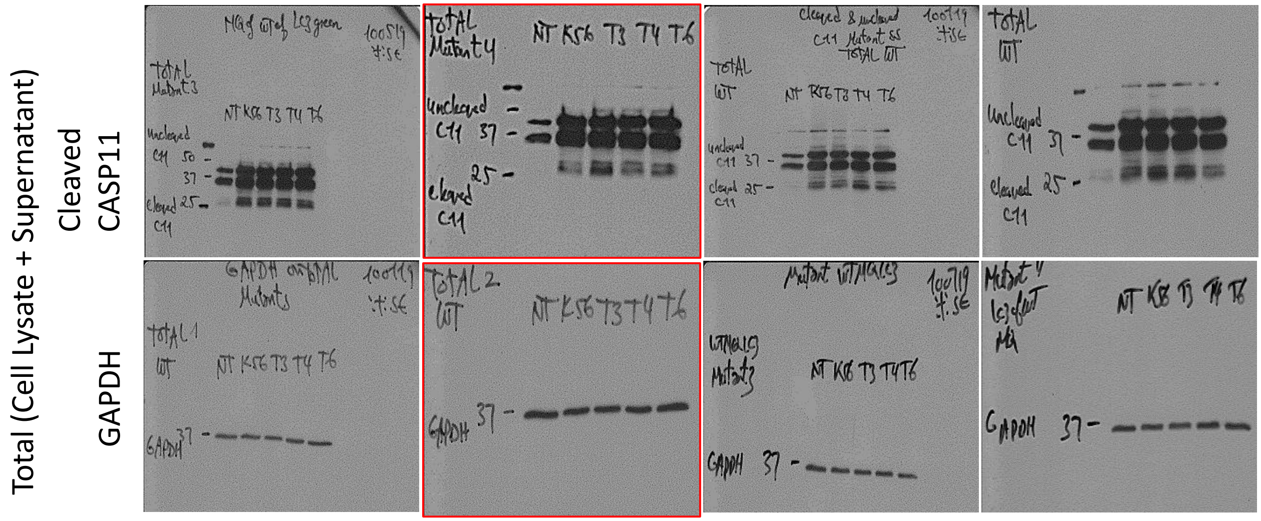

Figure 1D CASP1 N=4

Total (Cell Lysate + Supernatant)

CASP1

GAPDH

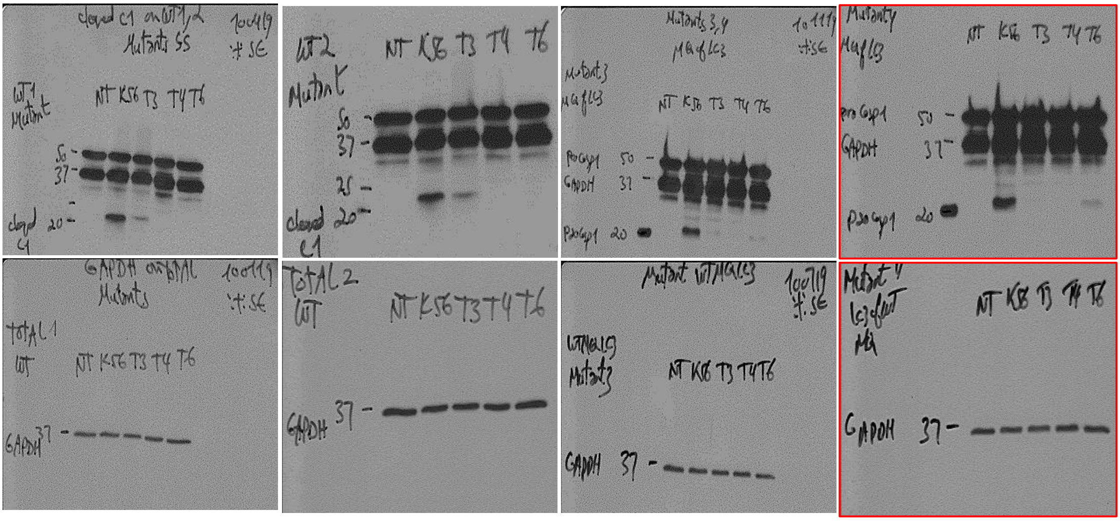


Figure 1D IL-1β N=4

Total (Cell Lysate + Supernatant)

IL-1β

GAPDH

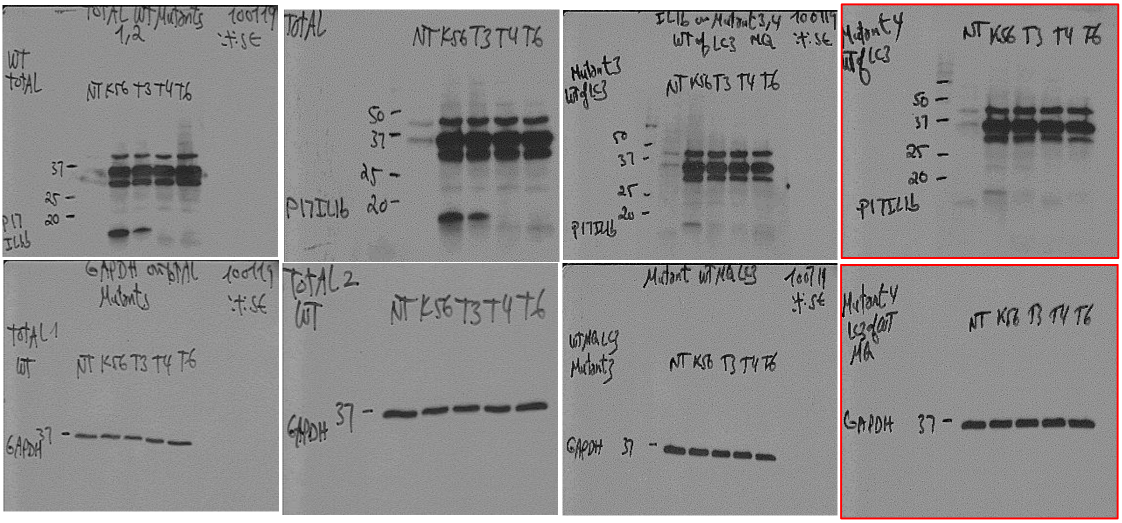


Figure 3C GAPDH Sup Without Glycine N=7

Cell Supernatant

GAPDH

GAPDH

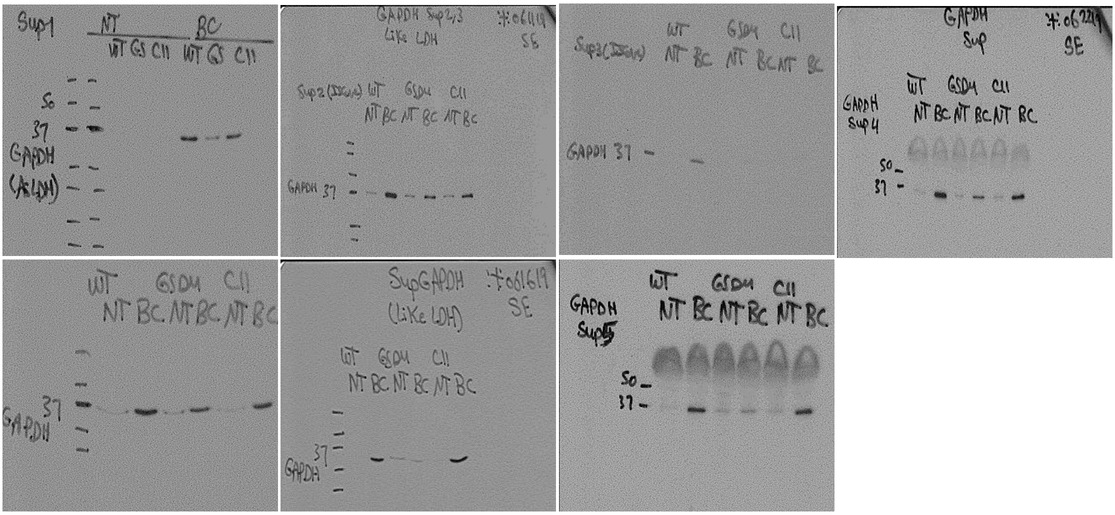

Figure 3C GAPDH Sup With Glycine N=6

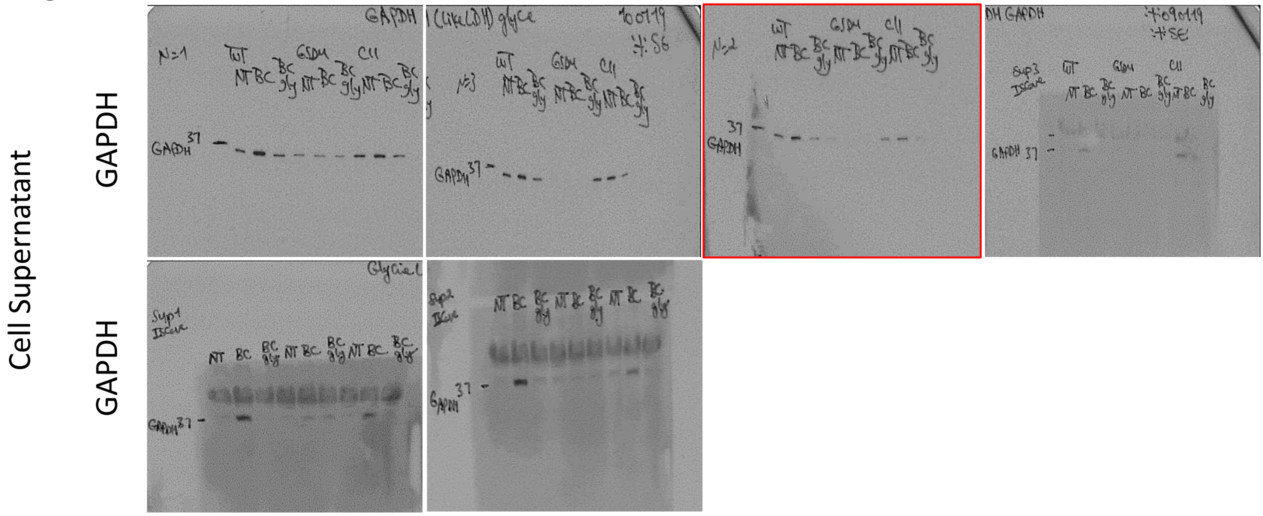


Figure 3C CASP1 Sup With Glycine N=3

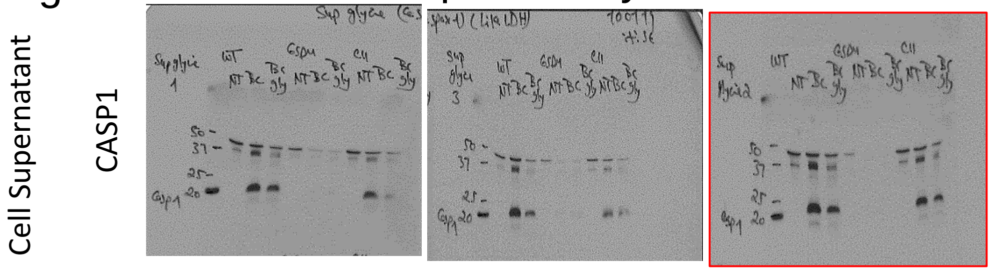


Figure 3C IL-1β Sup With Glycine N=3

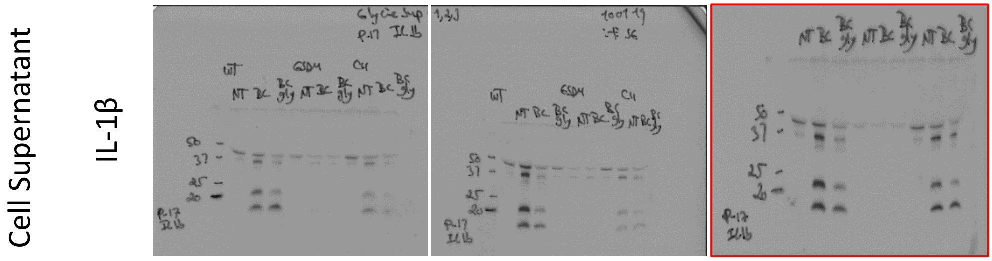


Figure 3C GSDMD Lysate With Glycine N=3

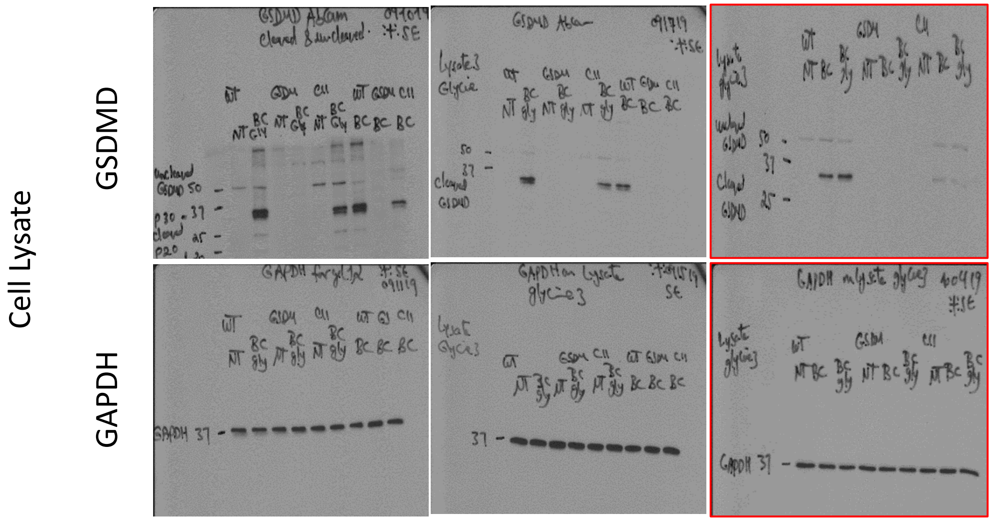

## Figure 3C CASP1 Lysate With Glycine N=3

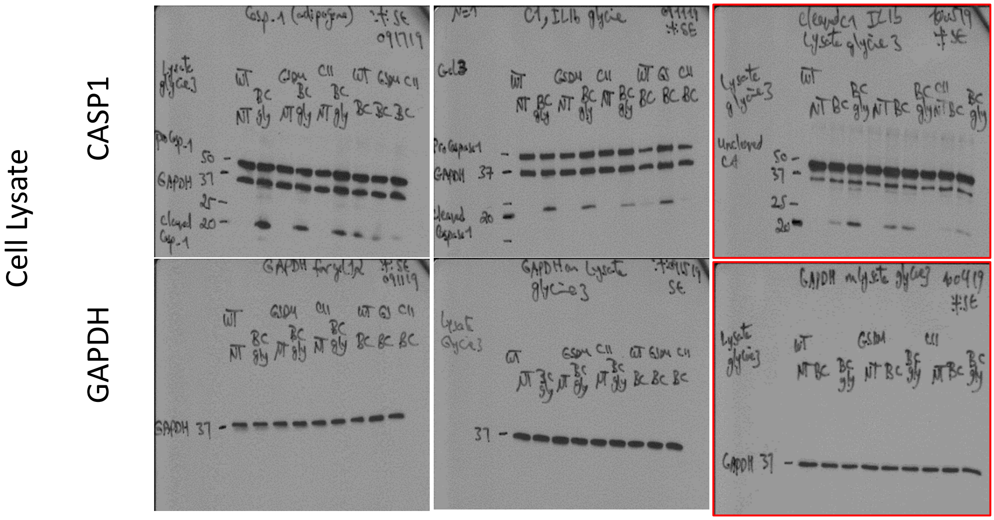


## Figure 3C IL-1β Lysate With Glycine N=3

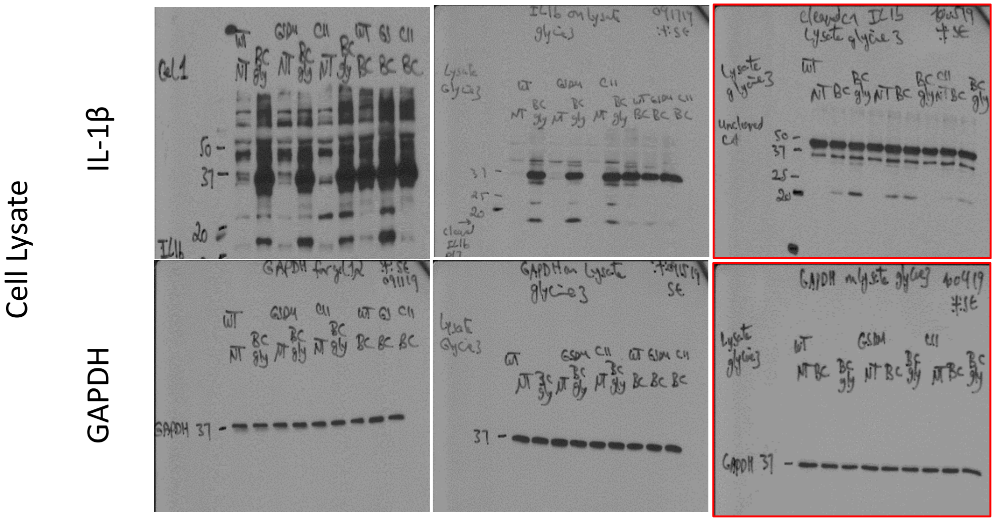

## Figure 3C CASP11 Lysate With Glycine N=3

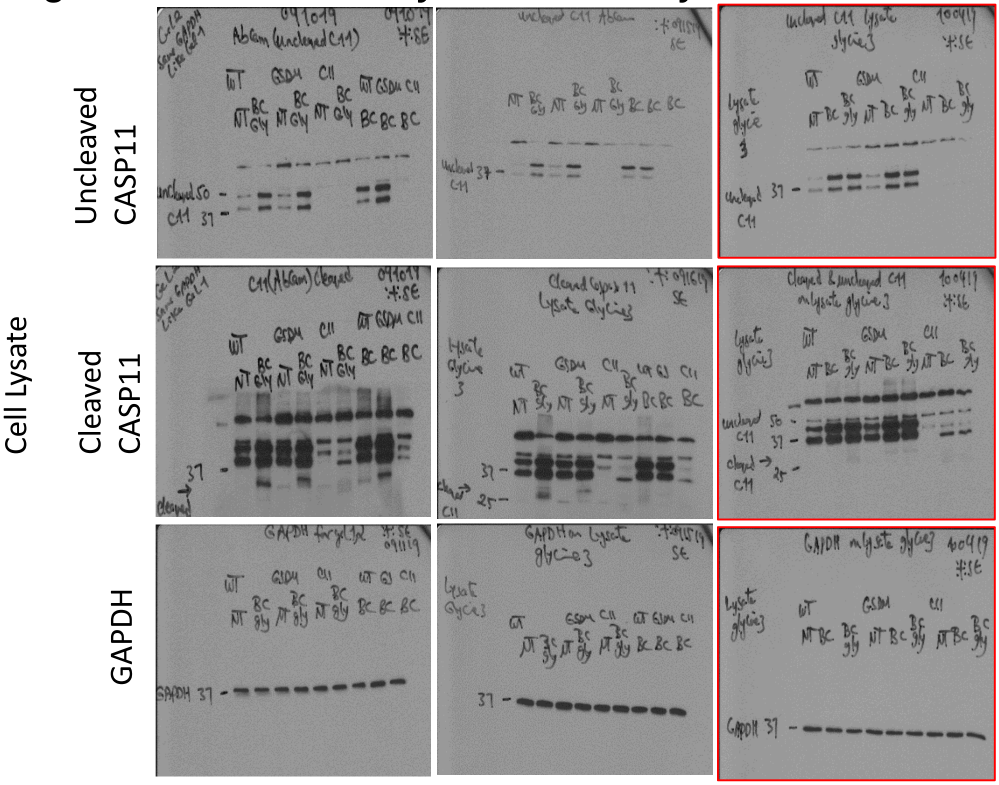


## Figure 5F

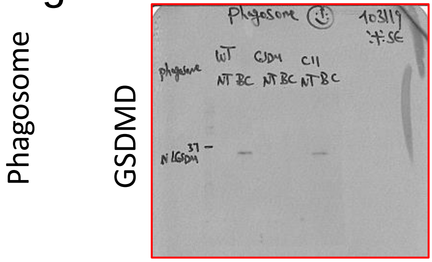


## Figure 6D N=4

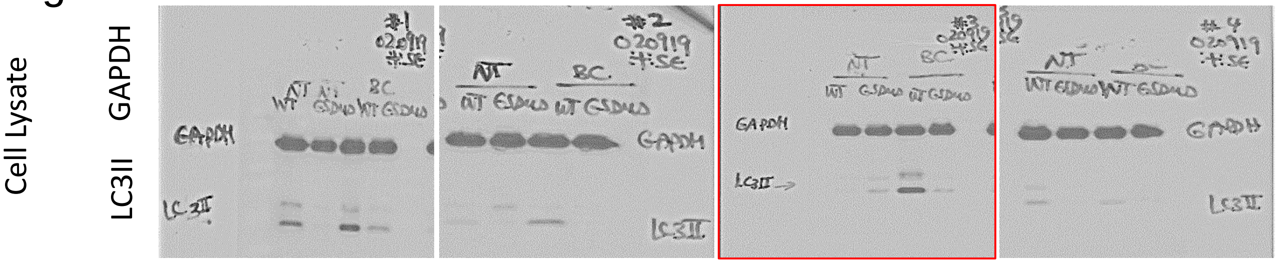


## Figure 6E

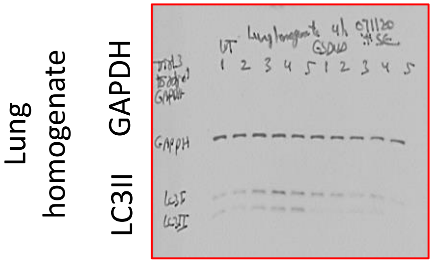

# Full-length gels and blots of the cropped blots in the supplementary figures

## Figure S1A N=5

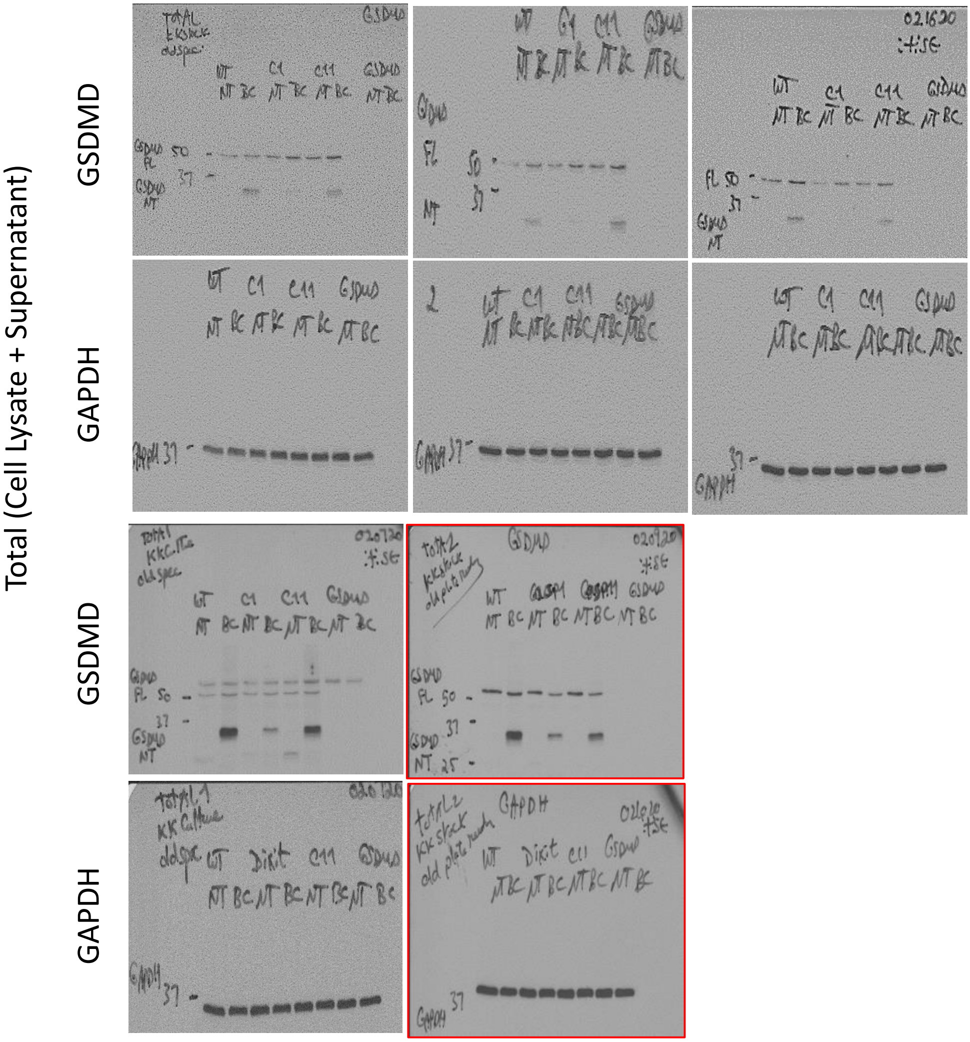


## Figure S5A

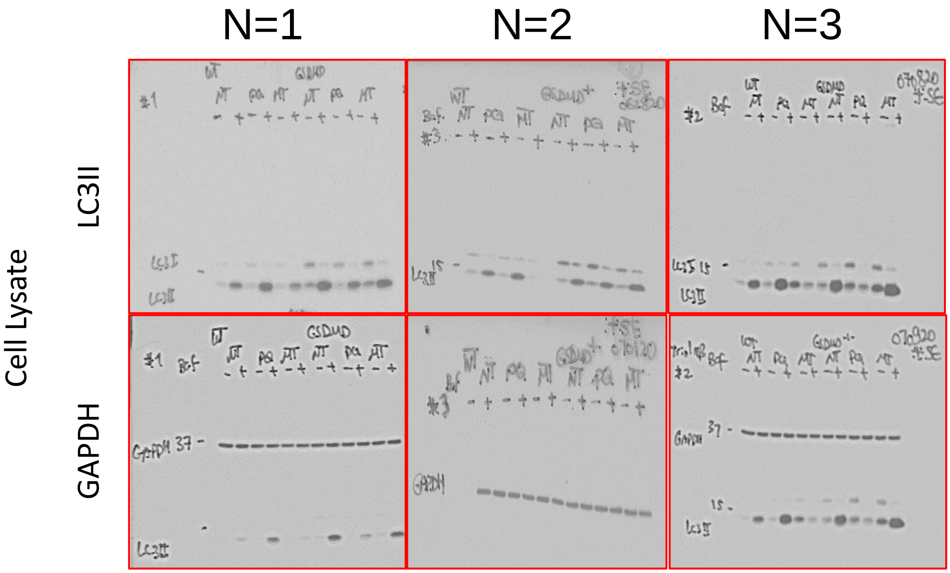

Supplement: Supplementary file 1 — Supplementary Information. [file 41598_2020_79201_MOESM1_ESM.pdf]
